# Toward a Molecular Framework of Systemic Multi-Organ Toxicity Induced by Chronic Aluminum Chloride Exposure

**DOI:** 10.3390/molecules31101728

**Published:** 2026-05-19

**Authors:** Ahmed S. A. Ali Agha, Sara Khaleel, Hamada M. A. Abdelaziz, Muhammed Alzweiri, Nidal A. Qinna, Ghayda’ AlDabet, Thaqif El Khassawna, Talal Aburjai

**Affiliations:** 1 School of Pharmacy, Department of Pharmaceutical Sciences, The University of Jordan, Amman 11942, Jordan; ahm9220505@ju.edu.jo (A.S.A.A.A.); hma9220006@ju.edu.jo (H.M.A.A.); m.alzweiri@ju.edu.jo (M.A.); thaqif.elkhassawna@chiru.med.uni-giessen.de (T.E.K.); 2 Department of Pharmacy, Faculty of Pharmacy, Al-Zaytoonah University of Jordan, Amman 11733, Jordan; s.malkawi@zuj.edu.jo; 3 Faculty of Pharmacy and Medical Sciences, University of Petra, Amman 11196, Jordan; nqinna@uop.edu.jo; 4 University of Petra Pharmaceutical Center (UPPC), Amman 11196, Jordan; ghayda.aldabet@uop.edu.jo; 5 Experimental Trauma Surgery, Faculty of Medicine, Justus-Liebig-University of Giessen, 35392 Giessen, Germany

**Keywords:** aluminum chloride (AlCl_3_), molecular mechanisms, metal-induced toxicity, oxidative stress, redox imbalance, mitochondrial dysfunction, molecular networks, systemic toxicity

## Abstract

Aluminum chloride (AlCl_3_) is widely used in experimental toxicology, particularly in rodent models of neurodegeneration, where its effects have been studied primarily in the central nervous system. However, experimental findings also indicate that chronic exposure is associated with changes across multiple peripheral organs, although these observations are often considered separately. In this review, we bring together evidence from different organ systems to examine aluminum toxicity from a broader perspective. Rather than focusing on isolated tissue-specific effects, we consider the extent to which reported findings may reflect overlapping molecular disturbances expressed across physiological systems. Within this context, organ-level outcomes are discussed as potentially related manifestations of shared underlying processes, while acknowledging variability in experimental conditions and model interpretation. To structure this synthesis, we outline a conceptual framework that links recurring molecular responses, system-level regulatory influences, and tissue-specific patterns of injury. This approach is intended to provide a more integrated way of organizing existing data rather than to establish a single unifying mechanism. Importantly, the pathological alterations discussed throughout this review are interpreted as experimentally observed toxicological manifestations of chronic AlCl_3_ exposure rather than evidence that aluminum constitutes a definitive etiological cause of Alzheimer’s disease. Overall, this review aims to complement existing neuro-focused interpretations of the AlCl_3_ model by situating it within a multi-organ context and highlighting areas where further integrative investigation may be warranted.

## 1. Introduction

Aluminum is one of the most abundant metals in the Earth’s crust and is widely present in food additives, pharmaceuticals, drinking water treatment processes, and numerous industrial products [1,2]. As a result, low-level human exposure to aluminum occurs routinely through environmental and dietary sources [3]. Although aluminum has no known biological function, chronic exposure has raised concerns about its potential toxicological and neurological effects due to its capacity to accumulate in biological tissues and disrupt cellular processes [4,5].

Among experimental approaches used to study aluminum toxicity, chronic administration of aluminum chloride (AlCl_3_) in rodents has become one of the most widely employed models in toxicological and neurodegenerative research [6]. In these experimental paradigms, aluminum exposure induces cognitive impairment, cholinergic dysfunction, oxidative stress, neuroinflammation, and neuronal degeneration—pathophysiological features commonly associated with Alzheimer’s disease (AD) [7,8]. In several animal studies, AlCl_3_ exposure has also been shown to increase acetylcholinesterase activity [9], promote amyloid-β accumulation [10], and enhance tau-related neuropathology [11], thereby producing AD-like behavioral and molecular changes in the brain. Consequently, the AlCl_3_ rat model has been widely adopted as a convenient experimental platform for investigating mechanisms of neurodegeneration and evaluating potential neuroprotective therapies.

Despite decades of research, the role of aluminum in the pathogenesis of Alzheimer’s disease remains unresolved and controversial [12,13]. Some studies have reported elevated aluminum concentrations in AD brain tissue, and several epidemiological studies, especially those examining aluminum in drinking water [13], have suggested possible associations with dementia risk. However, findings remain inconsistent and do not establish a causal relationship. Importantly, many experimental studies utilize controlled AlCl_3_ exposure paradigms to reproduce selected Alzheimer-like, neurotoxic, or organ-specific pathological features under defined toxicological conditions rather than to demonstrate aluminum as a definitive primary etiological cause of human AD. As summarized in Table 1, these experimental systems vary substantially in exposure dose, duration, administration route, and targeted biological outcomes across multiple organ systems.

Although chronic AlCl_3_ exposure is widely used in experimental neurotoxicity studies to reproduce selected Alzheimer-like molecular, biochemical, and behavioral features under controlled conditions, this use should be distinguished from its role in disease causation.

The primary scope of the present review is to examine the broader systemic toxicological consequences associated with chronic aluminum exposure across multiple organ systems. Accordingly, the alterations discussed throughout this review should be interpreted as experimentally observed toxicological manifestations of prolonged AlCl_3_ exposure rather than as evidence that aluminum is a definitive etiological cause of Alzheimer’s disease in humans.

Alzheimer’s disease is widely considered a multifactorial disorder involving interactions between genetic susceptibility, aging, environmental exposures, and metabolic disturbances [39]. Additional contributory factors reported in recent literature include microbiota-associated inflammation, blood–brain barrier impairment, and altered amyloid-β and tau homeostasis [40,41]. Nevertheless, growing interest in environmental contributors to neurodegeneration has renewed attention to aluminum as a possible modifying factor in disease development.

Beyond this etiological debate, another important reason to revisit the AlCl_3_ model lies in the distinction between Alzheimer-like neuropathology and general aluminum toxicity. While aluminum administration in experimental animals can reproduce several pathological features associated with AD, the experimental conditions across studies vary substantially in terms of dose, exposure duration, and route of administration [14]. Some studies employ relatively moderate chronic doses intended to induce gradual neurodegenerative changes [15], whereas others utilize substantially higher doses that may produce broader toxic effects [42]. Such methodological variability complicates model interpretation and raises questions about its mechanistic relevance and translational applicability to human exposure scenarios.

Importantly, most studies employing the AlCl_3_ model have focused predominantly on the central nervous system, often overlooking the systemic distribution and toxicity of aluminum. Aluminum is not restricted to the brain after exposure; rather, it can accumulate in multiple tissues and organs due to its slow clearance and strong binding to biological molecules. Chronic aluminum exposure has been reported to affect organs such as the liver and kidneys through disruption of antioxidant defense systems, Nrf2-associated redox imbalance, mitochondrial and metabolic dysfunction, lipid peroxidation, inflammatory activation, and progressive histopathological injury [19,42]. Downstream pathological responses vary across tissues and include endoplasmic reticulum stress and metabolic collapse in the liver, fibrogenic extracellular matrix remodeling in the kidney, and degenerative cellular injury mediated by caspase-dependent apoptotic signaling [8,43].

Taken together, these observations suggest that aluminum toxicity may represent a systemic biological process rather than a brain-restricted phenomenon. However, peripheral organ involvement has received comparatively limited attention in studies employing the AlCl_3_ model, which has historically been framed primarily as a neurodegenerative disease model.

The present review aims to re-examine the widely used AlCl_3_ experimental model through a systemic, multi-organ framework of toxicity. By integrating evidence across central and peripheral tissues, this review proposes that aluminum exposure should be understood as a multi-layer biological process involving systemic distribution, shared molecular stress responses, and organ-specific pathological outcomes. Through this integrative perspective, the review highlights common mechanisms—including oxidative stress dysregulation, mitochondrial impairment, inflammatory signaling, and apoptosis—that may link aluminum-induced pathology across different organ systems.

## 2. Molecular Brain Pathology in Chronic Aluminum Chloride Exposure

Chronic AlCl_3_ exposure in rodents produces a consistent pattern of central nervous system injury that primarily affects the hippocampus and cerebral cortex, regions essential for learning, memory, and higher cognitive processing [16,44,45]. These structures represent one of the most sensitive targets of systemic aluminum toxicity, and experimental models repeatedly demonstrate neuronal degeneration, synaptic disruption, and inflammatory activation within these areas [17,46]. Histopathological studies commonly report pyramidal neuron loss, cytoplasmic vacuolation, and structural disorganization of hippocampal subfields together with cortical neuronal damage and reactive gliosis, establishing the anatomical basis for the cognitive impairment observed in exposed animals [47,48,49].

**Figure 1:**
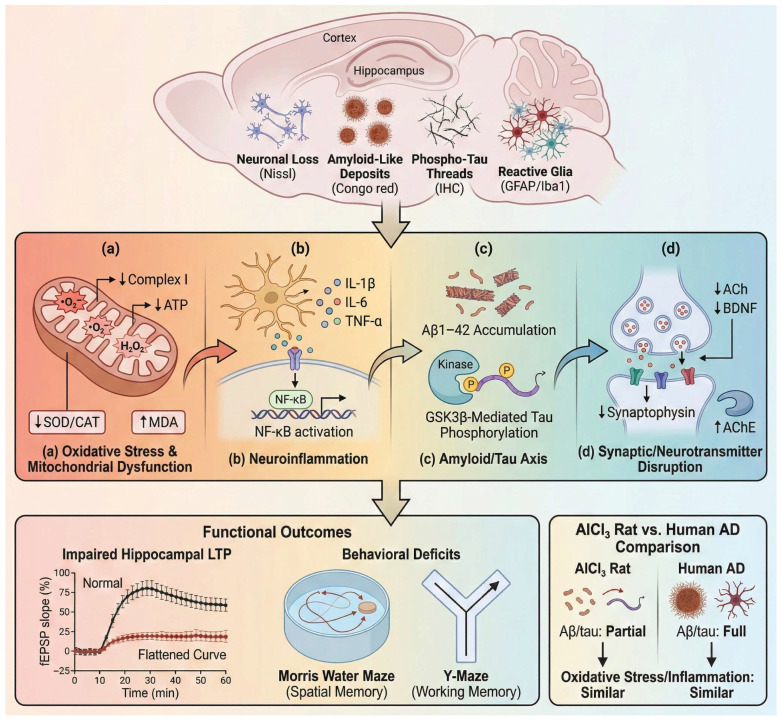
AlCl_3_ exposure is associated with hippocampal and cortical neuronal loss, amyloid-like deposits, phosphorylated tau (p-tau) changes, and reactive gliosis. Proposed mechanisms include oxidative stress and mitochondrial dysfunction, nuclear factor kappa B (NF-κB)–mediated neuroinflammation, amyloid-beta (Aβ_1–42_) accumulation with glycogen synthase kinase-3 beta (GSK3β)-dependent tau phosphorylation, and synaptic/neurotransmitter disruption involving acetylcholine (ACh), brain-derived neurotrophic factor (BDNF), synaptophysin, and acetylcholinesterase (AChE). These alterations are linked to impaired hippocampal long-term potentiation (LTP) and deficits in spatial and working memory tasks, while reproducing only partial amyloid/tau pathology compared with human AD.

Although the AlCl_3_ model has frequently been used to reproduce aspects of Alzheimer-like neurodegeneration, its phenotype is more accurately interpreted as a toxicologically induced neurodegenerative state rather than a full replication of human Alzheimer pathology [14]. Aluminum exposure promotes accumulation of amyloid-β species and increases tau phosphorylation, but the formation of mature plaques and neurofibrillary tangles remains inconsistent in wild-type rodents [4,50]. Consequently, the AlCl_3_ brain phenotype is best understood as an environmentally driven neurotoxicity model that captures several upstream molecular features shared with neurodegenerative disorders, particularly including ROS-mediated lipid peroxidation, NF-κB-dependent neuroinflammatory signaling, mitochondrial electron transport chain impairment, calcium dyshomeostasis, tau hyperphosphorylation, and synaptic dysfunction. Collectively, these processes promote neuronal apoptosis and cognitive decline, distinguishing the cerebral response from the ER stress–dominant hepatic pathology or fibrotic remodeling observed in renal tissue. As illustrated in Figure 1, these mechanisms converge on interconnected pathways linking oxidative injury, neuroinflammation, amyloid/tau dysregulation, and synaptic failure.

Representative molecular, cellular, and functional biomarkers used to characterize these pathological processes in experimental AlCl_3_ models are summarized in Table 2.

A central initiating mechanism is the induction of ROS-driven oxidative injury coupled with mitochondrial bioenergetic dysfunction. Chronic aluminum exposure promotes superoxide and hydrogen peroxide generation, lipid peroxidation, and peroxynitrite formation, while simultaneously impairing mitochondrial electron transport chain activity and depleting endogenous antioxidant defenses, including superoxide dismutase, catalase, glutathione, and glutathione peroxidase systems. These alterations are further amplified by iron-facilitated Fenton-like reactions, calcium dyshomeostasis, and disruption of tricarboxylic acid cycle metabolism, collectively driving neuronal oxidative damage and metabolic failure [57,58]. These redox disturbances are accompanied by impairment of mitochondrial bioenergetics and ATP production, creating a cellular environment that promotes neuronal vulnerability and initiates downstream injury cascades [59].

In parallel, oxidative stress promotes persistent neuroinflammation driven by activation of astrocytes and microglia. Aluminum exposure increases inflammatory mediators including interleukin-1β, interleukin-6, and tumor necrosis factor-α and activates NF-κB-dependent signaling pathways within hippocampal and cortical tissue [60]. This inflammatory environment further amplifies oxidative injury and contributes to neuronal degeneration, creating a feed-forward cycle linking redox imbalance with cytokine-mediated tissue damage.

Disturbances in amyloid and tau signaling represent another component of this pathological network. Experimental studies show that chronic aluminum exposure increases cerebral amyloid-β levels and enhances tau phosphorylation through dysregulation of intracellular kinase pathways such as GSK3β [11]. Additional AlCl_3_-specific evidence indicates that amyloid accumulation may involve enhanced amyloidogenic processing, as AlCl_3_ increases BACE1 expression while reducing Aβ clearance-related proteins such as LRP1 and neprilysin [61].

The tau component appears to involve kinase–phosphatase imbalance. In d-galactose/AlCl_3_ models, reduced PP2A activity and increased GSK3β levels have been linked to tau hyperphosphorylation, hippocampal cytoarchitectural disruption, and AD-like neurodegenerative changes [62].

Mechanistically, hyperphosphorylated tau is relevant because it loses its normal microtubule-stabilizing function, thereby impairing cytoskeletal organization, axonal transport, and synaptic integrity. However, the amyloid/tau pathology in AlCl_3_ models should be interpreted cautiously. Although some studies report amyloid-like deposits, neuritic plaque-like changes, and tau-associated pathology, these findings generally reflect partial proteinopathy rather than the full spectrum of mature extracellular plaques and intracellular neurofibrillary tangles characteristic of advanced human Alzheimer’s disease [14]. Accordingly, the amyloid/tau axis in AlCl_3_ exposure is best interpreted as an upstream toxicological disturbance overlapping with selected Alzheimer-related mechanisms, rather than as evidence of complete Alzheimer’s disease pathology.

Beyond protein aggregation and inflammatory injury, aluminum exposure produces pronounced disruption of synaptic and neurotransmitter systems. Cholinergic dysfunction is particularly consistent, with increased acetylcholinesterase activity and reduced acetylcholine availability frequently reported in hippocampal and cortical tissue [7]. Concurrent reductions in brain-derived neurotrophic factor and synaptic structural proteins further indicate compromised synaptic maintenance and plasticity, linking molecular injury to impaired neuronal communication.

These converging disturbances ultimately manifest as deficits in cognitive performance. Aluminum-exposed rodents consistently exhibit impairments in spatial and working memory in behavioral paradigms such as the Morris water maze and Y-maze, reflecting disruption of hippocampal circuitry [17]. Electrophysiological studies further demonstrate suppression of hippocampal long-term potentiation, a key cellular mechanism underlying memory formation, providing a functional correlate to the structural and molecular abnormalities observed following aluminum exposure [63].

Collectively, these findings support the utility of the AlCl_3_ model for studying selected neurodegeneration-associated molecular and behavioral alterations under controlled experimental conditions. However, the observed cerebral pathology should be interpreted within the broader context of toxicologically induced neurodegeneration rather than as a complete replication of human Alzheimer’s disease.

## 3. Molecular Mechanisms of Hepatic Injury in Chronic Aluminum Chloride Exposure

**Figure 2:**
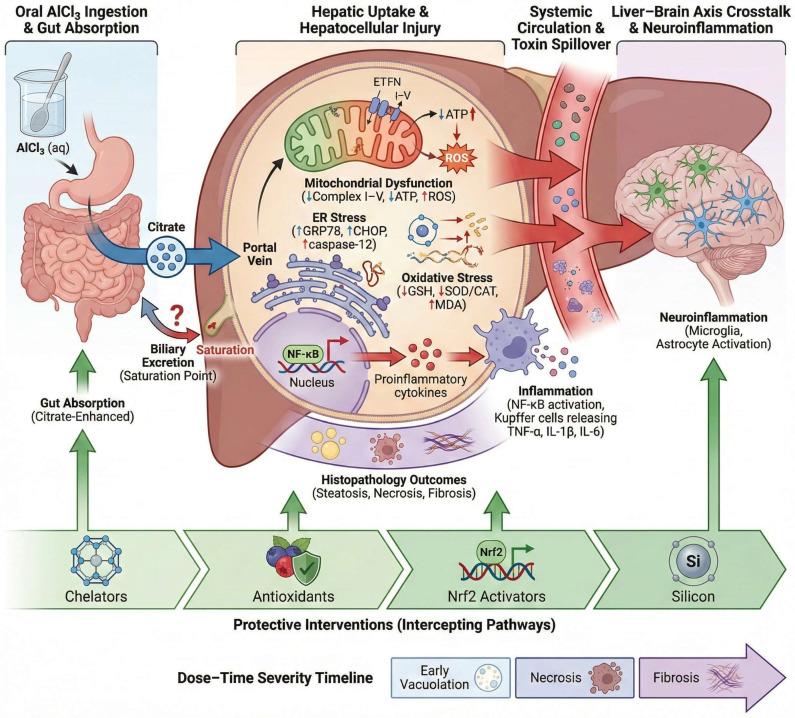
Conceptual schematic of molecular mechanisms underlying AlCl_3_-induced hepatic injury and systemic propagation. Illustration of the proposed pathways linking oral AlCl_3_ exposure to hepatocellular injury and downstream systemic effects. Following gastrointestinal absorption and portal delivery, aluminum accumulates in hepatocytes, where it induces mitochondrial dysfunction, oxidative stress (increased ROS and lipid peroxidation with depletion of antioxidant defenses), and endoplasmic reticulum stress, leading to activation of inflammatory signaling pathways (e.g., NF-κB) and production of proinflammatory cytokines. These processes contribute to hepatocellular damage and histopathological outcomes, including steatosis, necrosis, and fibrosis. Secondary systemic dissemination of inflammatory mediators and toxic signals may promote neuroinflammatory responses along the liver–brain axis. Potential intervention points—including chelators, antioxidants, Nrf2 activators, and silicon-based compounds—are indicated.

The liver is a major target of chronic AlCl_3_ exposure because orally absorbed aluminum reaches hepatocytes directly through the portal circulation, placing the liver at an early interface of toxicant handling and metabolic burden [42]. Experimental rodent studies show that prolonged AlCl_3_ exposure induces marked hepatocellular injury [42]. These alterations are associated with increased lipid peroxidation, depletion of antioxidant reserves, inhibition of membrane-associated enzymes, disruption of carbohydrate metabolism, and oxidative DNA damage. Importantly, although AlCl_3_ administration is frequently used experimentally to reproduce selected neurodegenerative or systemic pathological features under controlled conditions, the hepatic alterations discussed here reflect secondary toxicological manifestations of chronic aluminum exposure rather than intentional liver-specific disease modeling. Within this context, the liver serves not only as a primary site of aluminum accumulation and injury but also as a potential contributor to broader systemic metabolic and redox disturbances, as schematized in Figure 2.

Histopathologically, chronic AlCl_3_ exposure produces a reproducible hepatic injury pattern characterized by hepatocyte degeneration, sinusoidal congestion and enlargement, disruption of hepatic cords, loss of discrete hepatocellular boundaries, and necroinflammatory injury accompanied by inflammatory infiltration and hepatocellular ballooning [20,42]. In prolonged oral exposure models, necroinflammatory grade and ballooning score serve as useful quantitative markers of chronic hepatic damage [20]. These structural lesions are paralleled by a biochemical and molecular injury profile marked by increased serum ALT, AST, ALP, and, in some chronic oral models, GGT, together with hyperbilirubinemia, reduced albumin and total protein, enhanced lipid peroxidation, suppression of antioxidant defenses, and activation of inflammatory and endoplasmic reticulum stress pathways, collectively indicating progressive loss of hepatic cellular and functional integrity [42,64].

At the molecular level, oxidative stress constitutes the principal mechanism underlying AlCl_3_-induced hepatotoxicity. Chronic aluminum exposure promotes excessive reactive oxygen species generation and hepatic lipid peroxidation, evidenced by elevated MDA levels alongside depletion of endogenous antioxidant defenses, including GSH, SOD, CAT, GST, and HO-1. This sustained redox imbalance is closely associated with suppression of Nrf2-mediated antioxidant signaling, mitochondrial dysfunction, and oxidative degradation of cellular macromolecules. The resulting oxidative microenvironment activates inflammatory pathways, characterized by increased TNF-α, IL-1β, and NF-κB expression, further amplifying hepatocellular injury. Simultaneously, AlCl_3_ exposure induces apoptotic signaling through cytochrome c release and caspase-3 activation, culminating in hepatocyte degeneration, inflammatory infiltration, sinusoidal disruption, and progressive hepatic necrosis [19,42,64]. This oxidative collapse is accompanied by metabolic distress, including altered activities of membrane-associated enzymes and enzymes involved in carbohydrate and energy metabolism, together with dysregulation of antioxidant signaling pathways such as Nrf2, indicating that AlCl_3_ toxicity extends beyond nonspecific oxidative injury to broader disruption of hepatocellular metabolic and redox homeostasis.

Emerging evidence also implicates endoplasmic reticulum stress in AlCl_3_-induced liver injury, as hepatic upregulation of BiP/GRP78, CHOP, and XBP1 has been reported in exposed rats, with their attenuation paralleling biochemical recovery and histologic improvement in an intervention model [64]. This ER-stress response is closely coupled to hepatocyte apoptosis, with increased caspase-3 expression and related pro-apoptotic signaling providing further evidence of programmed cell death during chronic AlCl_3_ hepatotoxicity [19]. Thus, the hepatocyte in this model is best understood as a site where oxidative stress, ER stress, inflammatory signaling, and apoptosis converge rather than as a compartment dominated by a single isolated pathway.

Inflammatory activation accompanies AlCl_3_-induced hepatic injury, as evidenced by upregulated hepatic expression of IL-1β, TNF-α, and MMP9 together with downregulation of Nrf2, linking oxidant stress to a transcriptional inflammatory response and matrix-remodeling signaling in the injured liver [65]. Accordingly, inflammation can be viewed not only as a consequence of hepatocellular stress, but also as a mechanism that reinforces and sustains hepatic injury.

These interconnected pathways also reveal potential points of therapeutic interception, including antioxidant reinforcement of hepatic redox defenses [66,67,68], pharmacologic activation of Nrf2-dependent cytoprotective signaling [19,69], and toxicokinetic strategies that reduce aluminum bioavailability or tissue accumulation, such as chelation-based approaches and silicon-mediated formation of relatively inert hydroxyaluminosilicate complexes [70,71].

However, the available evidence should be interpreted cautiously. Although several preclinical studies report partial protection against AlCl_3_-induced oxidative, inflammatory, apoptotic, and histopathological injury, these findings remain heterogeneous across compounds, doses, exposure durations, and target organs. Chelation strategies have stronger precedent in aluminum-overload settings, whereas silicon-based approaches are mainly supported by toxicokinetic plausibility and limited experimental evidence in mammalian chronic AlCl_3_ toxicity. Therefore, these interventions should currently be regarded as experimental or candidate strategies rather than consistently validated therapies across AlCl_3_-induced multi-organ toxicity.

## 4. Renal Mechanisms of Aluminum Chloride Toxicity

**Figure 3:**
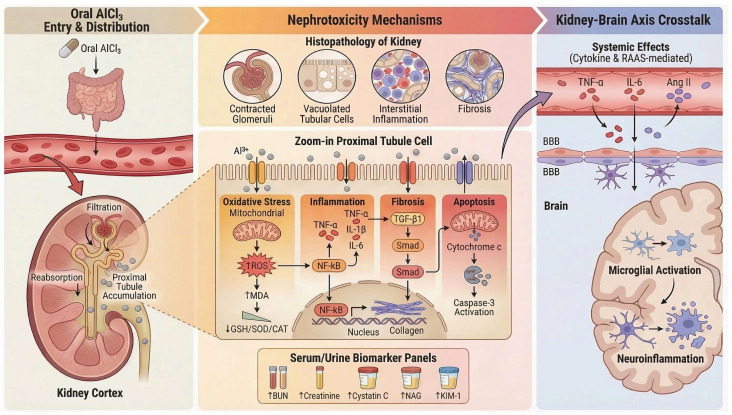
Conceptual overview of AlCl_3_-induced nephrotoxicity and kidney–brain axis interactions. Schematic representation of the proposed mechanisms linking oral AlCl_3_ exposure to renal injury and systemic effects. Following gastrointestinal absorption and systemic circulation, aluminum—often complexed with ligands such as citrate—is filtered at the glomerulus and accumulates in the renal cortex, particularly within proximal tubular epithelial cells. Intracellular aluminum promotes oxidative stress (increased ROS and lipid peroxidation with depletion of antioxidant defenses), leading to mitochondrial dysfunction, activation of inflammatory pathways (e.g., NF-κB, TNF-α, IL-1β, IL-6), apoptotic signaling (cytochrome c release, caspase-3 activation), and fibrogenic responses mediated in part by TGF-β/Smad signaling. These molecular events correspond to histopathological features including tubular degeneration, interstitial inflammation, glomerular alterations, and progressive fibrosis, alongside elevations in renal injury biomarkers (e.g., BUN, creatinine, cystatin C, KIM-1, NAG). Systemically, inflammatory mediators and dysregulated signaling may contribute to kidney–brain axis interactions, including microglial activation and neuroinflammation.

The kidney represents a major target of chronic AlCl_3_ exposure because renal excretion constitutes the primary route of aluminum elimination. Following systemic absorption, circulating aluminum—often complexed with low–molecular–weight ligands such as citrate—can be filtered at the glomerulus and delivered to the renal tubules, where preferential accumulation occurs within the renal cortex, particularly in proximal tubular epithelial cells [72]. This cortical localization reflects the kidney’s dual role as both a filtration interface and a site of solute concentration, rendering tubular cells especially susceptible to toxicant-induced injury [73]. The mechanistic framework linking aluminum accumulation to renal cellular injury and systemic consequences is summarized in Figure 3.

Histopathological studies of aluminum-induced nephrotoxicity describe a phenotype characterized by tubulointerstitial injury and oxidative stress. Excess ROS promotes lipid peroxidation and inflammatory responses, contributing to the degeneration of renal tubular epithelial cells and interstitial inflammatory infiltration. This injury disrupts normal renal architecture and is commonly accompanied by glomerular abnormalities such as vascular congestion, irregular urinary space, and glomerular atrophy [21]. With sustained exposure, renal tissue may exhibit increased extracellular matrix deposition and interstitial fibrosis, alongside tubular and interstitial injury, suggesting a shift toward more persistent structural remodeling. These histopathological changes are accompanied by elevated serum urea and creatinine levels, consistent with impaired renal function [22].

Contemporary nephrotoxicity research further identifies cystatin C, kidney injury molecule-1 (KIM-1), neutrophil gelatinase-associated lipocalin (NGAL), and urinary N-acetyl-β-D-glucosaminidase as sensitive biomarkers of early tubular injury [74]. Although these markers have not yet been systematically characterized across all AlCl_3_ nephrotoxicity studies, their mechanistic relevance aligns with the tubular injury profile observed in experimental models.

At the molecular level, oxidative stress represents the dominant driver of aluminum-induced nephrotoxicity. Renal exposure to AlCl_3_ promotes excessive reactive oxygen species generation and membrane lipid peroxidation, accompanied by depletion of glutathione and suppression of major antioxidant defenses, including SOD and CAT, thereby disrupting renal redox homeostasis. Mechanistically, aluminum impairs NADPH-generating pathways required for glutathione regeneration, enhances iron-mediated oxidative membrane injury, and compromises mitochondrial and microsomal detoxification capacity, resulting in hydrogen peroxide accumulation and progressive oxidative damage. This redox imbalance is further associated with tubular epithelial degeneration, glomerular collapse, inflammatory cell infiltration, cystic tubular dilation, and interstitial fibrotic remodeling, collectively contributing to renal dysfunction and chronic nephrotoxic progression [23]. This oxidative burden contributes to mitochondrial dysfunction and activates inflammatory signaling cascades characterized by increased IL-6, TNF-α, IL-1β, and NF-κB activity. Concurrently, aluminum exposure promotes cytochrome c release, Fas/FasL pathway activation, and BAX/caspase-3–mediated apoptosis, while suppressing antiapoptotic Bcl-2 signaling. In parallel, oxidative and inflammatory crosstalk upregulates MMP-9 expression, enhancing extracellular matrix degradation, epithelial–mesenchymal transition, and interstitial fibrotic remodeling within renal tissue [22]. Sustained oxidative and inflammatory stress promotes apoptotic signaling and extracellular matrix remodeling, processes that ultimately drive fibrotic transformation of the tubulointerstitial compartment. Fibrogenic mechanisms involving transforming growth factor-β-dependent signaling and matrix deposition are widely recognized contributors to toxicant-induced chronic kidney injury and provide a mechanistic basis for the progressive renal remodeling observed in aluminum exposure models [24].

Intervention studies further support this mechanistic framework. Compounds with antioxidant and anti-inflammatory properties, such as hesperidin, attenuate aluminum-induced renal injury by reducing oxidative stress, improving antioxidant capacity, modulating inflammatory responses, and limiting apoptosis and fibrotic remodeling [22]. Similar protective mechanisms are widely observed in experimental nephrotoxicity models in which restoration of redox balance and suppression of inflammatory cascades lead to improved renal function and structural preservation [75,76].

Beyond local renal toxicity, AlCl_3_ exposure in this model was associated with concurrent renal and brain injury, including impaired kidney function, renal histopathological damage, and neuronal inflammatory and degenerative changes, suggesting a broader systemic toxic effect involving both organs [21]. In the context of aluminum toxicity, where renal and neurological injury frequently occur within the same exposure paradigm, these observations raise the possibility of inter-organ interactions linking nephrotoxicity with increased susceptibility to neuroinflammatory injury.

Collectively, chronic AlCl_3_ exposure induces a renal phenotype characterized by cortical aluminum accumulation, tubulointerstitial injury, oxidative stress, inflammatory activation, apoptosis, and progressive fibrotic remodeling. Within the systemic framework of aluminum toxicity, the kidney therefore functions both as a primary site of toxicant injury and as a potential contributor to broader physiological dysregulation through impaired aluminum clearance and inflammatory signaling.

## 5. Molecular Pathways of Aluminum Chloride-Induced Cardiac Injury

Aluminum chloride exposure has increasingly been associated with cardiovascular injury characterized by cardiomyocyte redox disruption, mitochondrial respiratory impairment, and membrane instability. Experimental studies demonstrate that AlCl_3_ intoxication promotes extensive lipid and protein oxidation in cardiac tissue, reflected by elevated MDA, hydrogen peroxide, and protein carbonyl levels, together with depletion of glutathione-dependent antioxidant defenses. Mechanistically, aluminum exposure has been linked to impaired electron transport chain activity and excessive ROS generation, contributing to loss of membrane integrity, leakage of cardiac enzymes such as LDH and CK, and disruption of myocardial metabolic homeostasis. In parallel, AlCl_3_-induced alterations in LDL-C/HDL-C and TC/HDL-C ratios suggest concurrent disturbances in cardiac-associated lipid handling and atherogenic remodeling [77]. These alterations indicate a marked shift toward a pro-oxidant intracellular environment, compromising cardiomyocyte structural integrity and promoting myocardial injury.

Consistent with this oxidative imbalance, AlCl_3_ exposure suppresses the activity of key antioxidant enzymes, including superoxide dismutase, catalase, and glutathione peroxidase, thereby weakening the intrinsic antioxidant defense system of cardiomyocytes [25,77]. Recent histological work further shows that this redox disturbance is accompanied by increased cardiac nitric oxide and TNF-α, reduced catalase activity, and a transition from biochemical oxidative injury to overt tissue pathology, including myocyte degeneration, cytoplasmic vacuolation, vascular congestion, and inflammatory cell infiltration [78]. Taken together, these findings support an oxidative–inflammatory injury axis rather than a purely redox-limited lesion.

Biochemical evidence of myocardial damage is further supported by alterations in circulating cardiac biomarkers. AlCl_3_ administration significantly increases plasma levels of lactate dehydrogenase and creatine kinase while reducing their activity within cardiac tissue, reflecting membrane destabilization and enzyme leakage from damaged cardiomyocytes [25,77]. In parallel, disturbances in lipid metabolism are repeatedly reported, including increased total cholesterol, triglycerides, and LDL concentrations together with reduced HDL levels, indicating a dyslipidemic shift that likely interacts with oxidative myocardial injury rather than representing an isolated metabolic abnormality. In this sense, lipid peroxidation and systemic dyslipidemia appear to form interconnected components of the broader cardiovascular response to aluminium toxicity.

Beyond degeneration and enzyme leakage, available evidence now supports a genuine apoptotic component in the AlCl_3_ cardiac phenotype. In addition to histological evidence of myocyte injury, AlCl_3_ exposure increases the number of active caspase-3–positive cardiomyocytes, indicating activation of programmed cell death pathways within myocardial tissue [78]. The same study also demonstrated a significant increase in myocardial collagen deposition, supporting progression from acute cellular injury toward interstitial fibrotic remodeling [78]. These observations substantially strengthen the interpretation of AlCl_3_ cardiotoxicity as a remodeling process involving not only oxidative injury but also apoptosis and extracellular matrix expansion.

A particularly interesting extension of this pathology is the reported reduction in cardiac telocytes, identified as CD117-positive interstitial cells with small cell bodies and long cytoplasmic processes [78]. Telocytes are specialized stromal cells characterized by elongated telopodes and are proposed to participate in myocardial structural organization, intercellular signaling, and tissue homeostasis. Previous cardiac studies have also suggested potential roles in regenerative and reparative processes following myocardial injury.

In the AlCl_3_ model, telocyte reduction occurred alongside increased cardiomyocyte apoptosis, inflammatory infiltration, collagen deposition, and myocardial degeneration [78]. This association suggests that aluminum-induced cardiac injury may involve disruption of stromal-supportive cellular networks in addition to direct cardiomyocyte toxicity. However, because AlCl_3_-specific evidence is currently limited and mainly based on CD117 immunoreactivity, further studies using ultrastructural confirmation and multi-marker phenotyping are required before assigning a causal role to telocyte loss in aluminum-induced cardiotoxicity.

Functional cardiac alterations have also been documented. Electrocardiographic analyses in AlCl_3_-exposed animals reveal abnormalities in cardiac electrical activity, including altered QRS morphology and conduction disturbances, suggesting that structural myocardial injury can translate into measurable electrophysiological dysfunction. Complementary developmental evidence further supports the cardiotoxic potential of aluminium compounds: in zebrafish larvae, exposure to AlCl_3_ induces pericardial oedema and significant reductions in heart rate, indicating direct impairment of cardiac physiology during early developmental stages [79].

**Figure 4:**
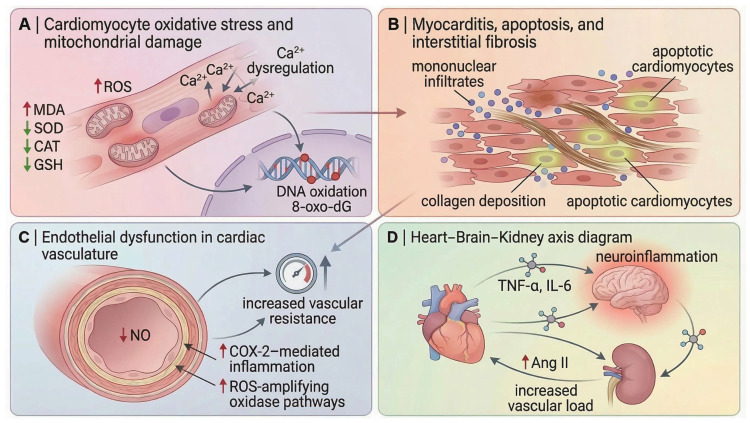
Conceptual schematic of molecular and structural mechanisms underlying AlCl_3_-induced cardiac injury and systemic interactions. Schematic illustration of the proposed pathways linking AlCl_3_ exposure to myocardial injury. (**A**) Cardiomyocyte oxidative stress characterized by increased ROS and lipid peroxidation, depletion of antioxidant defenses, mitochondrial dysfunction, Ca^2+^ dysregulation, and oxidative DNA damage. (**B**) Progression to structural myocardial pathology, including inflammatory cell infiltration, cardiomyocyte apoptosis, and interstitial fibrosis with collagen deposition. (**C**) Endothelial dysfunction within the cardiac vasculature, marked by reduced nitric oxide bioavailability, increased oxidative signaling, and pro-inflammatory activation, contributing to elevated vascular resistance. (**D**) Potential systemic consequences through heart–brain–kidney axis interactions, involving circulating inflammatory mediators (e.g., TNF-α, IL-6) and neuroinflammatory responses.

Taken together, current evidence indicates that AlCl_3_-mediated cardiotoxicity arises from a convergent network of oxidative injury, depletion of antioxidant defenses, inflammatory activation, dysregulated lipid metabolism, enzymatic leakage from damaged cardiomyocytes, apoptosis, and progressive interstitial remodeling. The emerging reduction in cardiac telocyte abundance further suggests that aluminum toxicity may impair not only myocardial survival but also myocardial repair capacity. Within the broader framework of systemic aluminum toxicity, these mechanisms position the heart as both a direct target of toxic injury and a potential contributor to wider vascular and neurobiological dysfunction. The principal molecular and structural pathways involved in aluminum-induced cardiac injury are summarized in Figure 4.

## 6. Molecular and Cellular Mechanisms of Aluminum Chloride-Induced Pulmonary Toxicity

**Figure 5:**
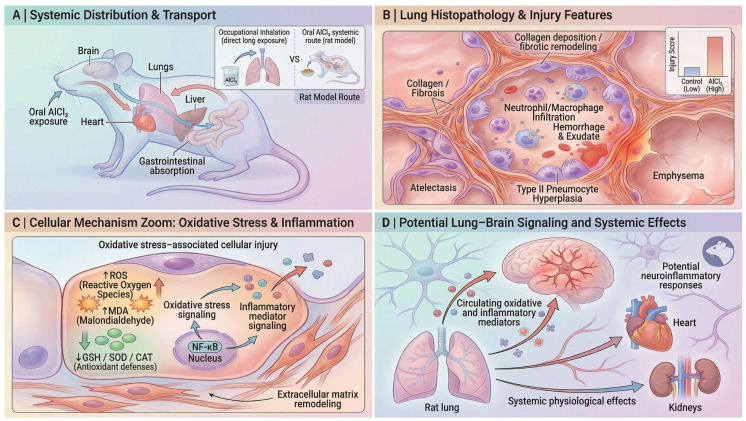
Conceptual overview of systemic distribution, pulmonary injury, and potential lung–brain interactions in AlCl_3_ exposure. Schematic representation of the proposed mechanisms underlying pulmonary effects of AlCl_3_ following systemic absorption. (**A**) Oral exposure leads to gastrointestinal absorption and systemic distribution of aluminum to peripheral organs, including the lungs. (**B**) Histopathological features of lung injury include alveolar collapse, interalveolar septal thickening, vascular congestion, hemorrhage, inflammatory cell infiltration, and collagen deposition consistent with early fibrotic remodeling. (**C**) At the cellular level, aluminum induces oxidative stress characterized by increased reactive oxygen species and lipid peroxidation, depletion of antioxidant defenses, activation of inflammatory signaling pathways (e.g., NF-κB), and extracellular matrix remodeling. (**D**) Potential systemic consequences include the release of circulating oxidative and inflammatory mediators, which may contribute to inter-organ signaling, including possible lung–brain interactions and neuroinflammatory responses.

Although most experimental AlCl_3_ models rely on oral administration rather than inhalational exposure, absorbed aluminum can distribute systemically and accumulate in peripheral organs including the lung. Consequently, pulmonary pathology observed in these models is generally interpreted as a secondary manifestation of systemic aluminum toxicokinetics rather than a primary airway deposition injury. Following oral exposure, aluminum is absorbed through the gastrointestinal tract and enters systemic circulation, allowing distribution to multiple organs such as the lung, liver, heart, and brain (Figure 5A).

Toxicokinetic studies further indicate that aluminum particles may persist within pulmonary tissues due to relatively slow clearance by alveolar macrophages, highlighting the lung as a potential target organ for aluminum toxicity [80].

Experimental studies suggest that circulating aluminum can affect pulmonary tissue through systemic transport, with the lung epithelium representing a potential target site for metal-induced injury [27,81].

Rodent studies demonstrate that chronic AlCl_3_ exposure produces substantial structural alterations within the alveolar compartment. Histological examinations commonly reveal collapse of alveolar spaces, thickening of interalveolar septa, vascular congestion, and extravasation of erythrocytes, indicating disruption of the alveolar–capillary barrier and increased vascular permeability [27,82]. These lesions are frequently accompanied by infiltration of inflammatory cells within alveolar and interstitial regions. Hemosiderin-laden macrophages may also be present within alveolar spaces, reflecting phagocytosis of erythrocyte degradation products following hemorrhagic events [81].

Comparable pulmonary alterations have also been described in aluminum-treated Wistar rats, where congestion of pulmonary blood vessels, alveolar hemorrhage, and distortion of alveolar architecture were observed, indicating substantial damage to pulmonary parenchyma [83]. Additional parenchymal alterations include emphysema-like enlargement of airspaces, interstitial edema, and inflammatory infiltration, indicating disruption of alveolar septal structures and connective tissue integrity [81,84]. Injury may also extend to bronchiolar epithelium, where epithelial desquamation and luminal debris have been reported [84].

Developmental exposure studies further demonstrate that postnatal lung structure may be affected by aluminum toxicity, with reports of bronchial epithelial destruction, intra-bronchiolar hemorrhage, and pronounced thickening of interalveolar septa in AlCl_3_-exposed rats [85]. These characteristic pulmonary lesions are summarized schematically in Figure 5B.

Ultrastructural studies further demonstrate cellular damage within lung tissue following aluminum exposure. Transmission electron microscopy reveals degeneration of pneumocytes, particularly type II pneumocytes, which are involved in surfactant synthesis and alveolar repair [27]. Observed abnormalities include nuclear pyknosis, chromatin condensation, cytoplasmic vacuolization, organelle loss, swollen mitochondria with disrupted cristae, and degenerative changes in surfactant-associated lamellar bodies [27].

These alterations suggest impaired alveolar epithelial metabolism and surfactant function. Increased numbers of type II pneumocytes following AlCl_3_ exposure likely reflect a compensatory regenerative response to epithelial injury [27,82].

A central mechanism underlying these pulmonary alterations is the disruption of the alveolar redox environment, driven by excessive ROS production and impaired mitochondrial oxidative phosphorylation [81]. Aluminum exposure markedly elevates pulmonary malondialdehyde (MDA), hydrogen peroxide (H_2_O_2_), and protein carbonyl levels, indicating oxidative degradation of membrane lipids and structural proteins within the alveolar epithelium. In the lung, this redox imbalance is closely associated with increased vascular permeability, elastic fiber degeneration, and epithelial barrier disruption, promoting alveolar edema and emphysematous remodeling [81]. Concurrent accumulation of mast cells and hemosiderin-laden macrophages further suggests that oxidative injury is coupled to inflammatory activation and microvascular damage within the pulmonary architecture [81]. Non-enzymatic antioxidant reserves—including reduced glutathione (GSH), non-protein thiols (NPSH), and vitamin C—are also significantly reduced, reflecting increased consumption of antioxidant molecules in response to reactive oxygen species generation [81]. In contrast, metallothionein levels increase in aluminum-treated lungs, suggesting a compensatory response to oxidative stress and metal exposure. These oxidative alterations and associated cellular processes are illustrated conceptually in Figure 5C.

Similar oxidative disturbances have also been reported in rats co-exposed to aluminum and acrylamide, where increased MDA, hydrogen peroxide, and advanced oxidation protein products (AOPP) were accompanied by depletion of glutathione and evidence of DNA fragmentation in lung tissue [26].

Oxidative stress–mediated injury also disrupts pulmonary membrane and alveolar barrier integrity, potentially amplified by aluminum-induced mitochondrial dysfunction and disturbed iron homeostasis that enhance ROS generation in lung tissue. Consistent with this mechanism, decreased lung LDH activity alongside elevated plasma LDH levels indicates epithelial membrane leakage and cellular injury, contributing to inflammatory infiltration, vascular permeability, and emphysematous remodeling [81].

Histological analyses demonstrate infiltration of inflammatory cells within thickened interalveolar septa and perivascular regions [82]. Mast cell accumulation has also been reported, suggesting activation of innate immune responses and release of mediators that may influence vascular permeability and local inflammatory processes [82]. Macrophages present within alveoli frequently contain hemosiderin deposits, indicating phagocytic clearance of erythrocyte remnants and damaged cellular material [81]. Sustained oxidative stress and inflammation may subsequently promote structural remodeling of lung tissue. Histological evidence indicates increased deposition of collagen fibers within interalveolar septa and perivascular areas following aluminum exposure, suggesting activation of fibroblasts and early fibrogenic responses [82]. These changes contribute to thickened septal structures and altered alveolar architecture, as conceptually illustrated in Figure 5B.

Within the broader framework of systemic aluminum toxicity, pulmonary injury may also contribute to systemic signaling processes. The lung represents a large vascular interface that can release oxidative and inflammatory mediators into the circulation. Although direct experimental evidence demonstrating a lung–brain signaling pathway in the AlCl_3_ model remains limited, the coexistence of pulmonary oxidative stress and well-documented neurotoxic effects of aluminum suggests that circulating mediators generated during lung injury could potentially influence distant organs. As illustrated conceptually in Figure 5D, circulating oxidative and inflammatory mediators originating from injured lung tissue may contribute to systemic physiological effects and potentially influence neuroinflammatory responses in the central nervous system.

## 7. Aluminum Chloride-Induced Reproductive Toxicity: Mechanistic Overview

Chronic AlCl_3_ exposure produces a reproducible pattern of reproductive toxicity in experimental models, indicating that aluminum’s systemic effects extend beyond classical neurotoxicity to involve the gonads and the endocrine networks that regulate fertility [86]. Evidence from rodent studies demonstrates that both male and female reproductive systems are vulnerable to aluminum-induced injury, although the specific pathological manifestations differ between testes and ovaries. In both systems, however, oxidative stress, endocrine disruption, inflammatory signaling, and apoptotic cell loss emerge as recurring mechanistic drivers. Within the broader framework of systemic aluminum toxicity, the reproductive organs therefore represent sensitive peripheral targets in which redox imbalance, hormonal dysregulation, and structural tissue injury converge to impair reproductive competence.

### 7.1. Mechanisms of Male Reproductive Toxicity

Chronic AlCl_3_ exposure produces a consistent, multi-level pattern of male reproductive toxicity in rodents, involving endocrine, structural, and cellular mechanisms. In adult exposure models, AlCl_3_ suppresses testosterone, LH, and FSH, induces degeneration of seminiferous tubules with reduced Johnsen scores and epithelial thickness, and disrupts spermatogenesis alongside downregulation of key sperm structural proteins (AKAP4, ODF1, OAZ3) and mitochondrial/ribosomal regulators, indicating bioenergetic and translational dysfunction [28]. Complementary developmental evidence shows that prenatal exposure leads to delayed puberty, reduced sperm count, and persistent spermatogenic defects, mediated by Sertoli cell and blood–testis barrier dysregulation, including altered tight-junction proteins and cytoskeletal dynamics that impair germ-cell progression [87].

Together, these findings demonstrate that AlCl_3_ targets hormonal regulation, seminiferous epithelium integrity, sperm structural machinery, and testicular cellular energetics, supporting the view that male reproductive endpoints are among the most sensitive outcomes in rodent aluminum exposure.

Histopathologically, AlCl_3_ exposure produces a coherent pattern of testicular degeneration that directly translates into impaired sperm production and quality. Testes exhibit atrophy and degeneration of seminiferous tubules, with loss and disorganization of spermatogenic cells, reduced spermatid/sperm content, basement membrane thickening, and interstitial edema with inactive Leydig cells [29].

These structural lesions are quantitatively reflected by reduced tubule diameter, area, and germinal epithelial integrity, alongside sparse germ-cell layers and diminished intratubular spermatozoa [88]. Consistently, these histological changes correspond to functional deterioration in sperm output and quality, including reduced sperm count, impaired motility, increased abnormal sperm morphology, and decreased mitochondrial membrane potential [29,88]. At the molecular level, AlCl_3_-associated reproductive toxicity is characterized by oxidative disruption of spermatogenic and testicular cellular homeostasis, including lipid peroxidation, depletion of enzymatic (SOD, CAT, GPx, GR) and non-enzymatic (GSH, vitamins C and E) antioxidant defenses, impaired mitochondrial membrane potential and ATP production, and activation of germ-cell apoptosis and mitophagy pathways involving Bax/caspase signaling and PINK1/Parkin/LC3B-mediated mitochondrial turnover. These alterations are accompanied by seminiferous tubule degeneration, germinal epithelium detachment, reduced Ki-67 proliferative activity, Leydig-cell degeneration, and impaired reproductive hormone production [89,90,91].

Importantly, AlCl_3_ toxicity extends beyond the testis to the epididymis, further amplifying reproductive dysfunction. Experimental evidence demonstrates decreased epididymal weight, marked reductions in antioxidant enzymes, and increased lipid peroxidation in epididymal tissue, indicating disruption of the oxidative environment required for sperm maturation and storage [90]. Histologically, the epididymis exhibits epithelial disorganization, apoptotic cell accumulation, inflammatory infiltration, and luminal spermatid loss or clustering, consistent with impaired sperm maturation and transport [92,93]. These combined testicular–epididymal alterations mechanistically explain the observed sperm defects, including reduced viability, increased DNA fragmentation, and predominant head and flagellar abnormalities [92,93].

Chronic exposure further promotes tissue remodeling characterized by interstitial expansion, fibrosis, and mononuclear inflammatory-cell infiltration [93], linking oxidative stress to sustained structural damage. These alterations reinforce endocrine disruption, as Leydig-cell injury contributes directly to reduced testosterone levels and downstream hypothalamic–pituitary–gonadal axis imbalance [92,94].

**Figure 6:**
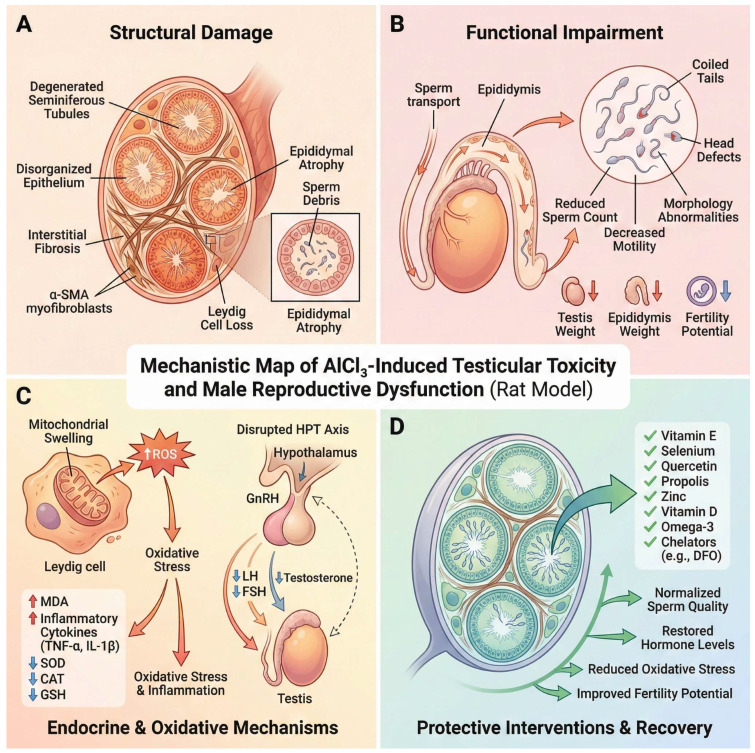
Conceptual overview of AlCl_3_-induced testicular toxicity and male reproductive dysfunction in rodents. (**A**) Structural damage: Chronic AlCl_3_ exposure is associated with degeneration of seminiferous tubules, disorganization of the germinal epithelium, Leydig-cell impairment, interstitial alterations including fibrosis, and epididymal atrophy with accumulation of sperm debris. (**B**) Functional impairment: These structural alterations are linked to reduced sperm count, impaired motility, increased morphological abnormalities (e.g., head and tail defects), decreased testicular and epididymal weights, and reduced fertility potential. (**C**) Endocrine and oxidative mechanisms: AlCl_3_ exposure is proposed to promote reactive oxygen species (ROS) generation and mitochondrial dysfunction, contributing to oxidative stress (e.g., increased lipid peroxidation and inflammatory cytokines, decreased antioxidant defenses) and disruption of the hypothalamic–pituitary–testicular (HPT) axis, with consequent reductions in testosterone, LH, and FSH levels. (**D**) Protective interventions: Antioxidant and supportive strategies (e.g., vitamin E, zinc, selenium, quercetin, omega-3 fatty acids, and chelators) have been reported to mitigate oxidative stress and partially restore hormonal balance, sperm quality, and fertility outcomes. This figure summarizes and integrates findings from experimental studies and represents a conceptual framework rather than a direct causal pathway.

Functionally, these lesions are accompanied by organ-level and cellular deficits, including reduced testicular and epididymal weights, depletion of germ-cell populations, and pronounced abnormalities in sperm morphology (e.g., head defects, coiled or bent tails, microcephaly, and structural deformities), reflecting impaired spermiogenesis and maturation [90,92,93]. Notably, part of this toxicity is partially reversible, as treatment withdrawal improves testicular histoarchitecture, germ-cell counts, and hormonal levels, while antioxidant-based interventions mitigate oxidative, inflammatory, and apoptotic damage [91,94]. Together, the evidence shows that AlCl_3_-induced histopathology is tightly coupled to functional spermatogenic failure, where coordinated disruption of endocrine signaling, seminiferous epithelium integrity, oxidative balance, epididymal function, and germ-cell survival directly manifests as compromised sperm production, quality, and fertility potential, as illustrated in Figure 6.

### 7.2. Mechanisms of Female Reproductive Toxicity

Prolonged aluminum chloride (AlCl_3_) exposure induces female reproductive toxicity through integrated endocrine, metabolic, ultrastructural, and histopathological mechanisms that progress with exposure duration. Under long-term subchronic conditions (e.g., 120 days), AlCl_3_ significantly suppresses ovarian endocrine function, evidenced by decreased circulating estradiol, progesterone, FSH, and LH, alongside increased ovarian aluminum accumulation and reduced ovarian weight, indicating disruption of the hypothalamic–pituitary–ovarian axis and impaired follicular maturation [95]. Consistently, shorter-duration experimental models demonstrated that AlCl_3_ administration reduces circulating FSH, LH, estradiol, and progesterone levels in female Wistar rats, accompanied by ovarian structural alterations including follicular degeneration and corpus luteum atresia [96].

At the biochemical level, AlCl_3_ interferes with steroidogenesis by reducing ovarian 3β- and 17β-hydroxysteroid dehydrogenase activities, increasing ovarian cholesterol, and decreasing estradiol synthesis, while concurrently disturbing uterine carbohydrate metabolism through glycogen accumulation and reduced phosphorylase activity [97]. In parallel, aluminum exposure disrupts ovarian redox homeostasis by increasing lipid peroxidation and depleting key antioxidant defenses, particularly GSH, SOD, and GPx, with more pronounced alterations observed at higher exposure doses and prolonged durations. Notably, GPx activity appeared especially sensitive to chronic aluminum exposure, showing reductions even at lower-dose long-term exposure, whereas catalase activity remained relatively preserved in this model [98].

These functional disturbances are supported by ultrastructural evidence of ovarian injury, including granulosa-cell apoptosis, chromatin margination, mitochondrial swelling and vacuolization, dilated endoplasmic reticulum, and Golgi disorganization, together with reduced activities of membrane-bound and mitochondrial enzymes (Na^+^/K^+^-, Ca^2+^-, Mg^2+^-ATPases, SDH, ACP, ALP), altered Fe/Zn/Cu homeostasis, and decreased expression of FSH and LH receptors, collectively indicating impaired bioenergetics, disrupted gonadotropin signaling, and defective ovulation and corpus luteum formation [99]. Correspondingly, histological analyses in AlCl_3_-exposed ovaries revealed degeneration and necrosis of follicular cells, increased numbers of atretic follicles, and regression of corpus luteum structures [96]. At the molecular level, AlCl_3_ further modulates ovarian gene expression, including dysregulation of Cyp19a1, Pcna, Puma, and Map1lc3b, reflecting altered steroidogenesis, reduced proliferation, and activation of apoptosis and autophagy pathways in granulosa cells [30]. The observed depletion of antioxidant enzymes in ovarian tissue further reflects disruption of the redox balance under aluminum exposure [98].

Concomitantly, subchronic exposure (up to 60 days) produces progressive histopathological alterations across the reproductive tract, including ovarian follicular degeneration and atresia, stromal congestion and fibrosis, and corpus luteum abnormalities, together with oviductal epithelial hyperplasia, papillary projections, inflammatory infiltration, and uterine lesions characterized by cystic glandular dilation, epithelial vacuolation, necrosis, and mucopurulent endometrial inflammation [31]. In agreement, uterine histology following AlCl_3_ exposure showed epithelial degeneration, vacuolation, glandular dilation, and endometrial thickening in female Wistar rats [100].

**Figure 7:**
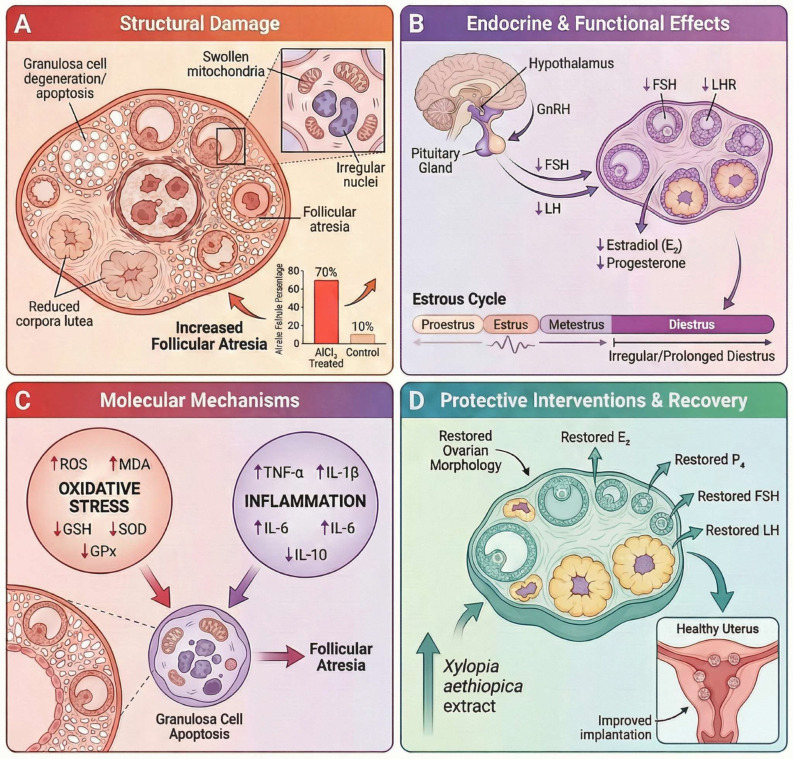
Conceptual mechanistic overview of aluminum chloride (AlCl_3_)-induced ovarian and uterine alterations in female rats. This schematic summarizes experimentally reported structural, endocrine, and molecular changes associated with AlCl_3_ exposure. (**A**) Ovarian structural alterations include granulosa cell degeneration/apoptosis, mitochondrial and nuclear abnormalities, reduced corpora lutea, and increased follicular atresia. (**B**) Endocrine effects involve disruption of the hypothalamic–pituitary–ovarian axis, reflected by decreased FSH, LH, estradiol, and progesterone levels, with associated disturbances in estrous cyclicity. (**C**) Proposed molecular mechanisms include oxidative stress (elevated ROS and lipid peroxidation with reduced antioxidant defenses, including GSH, SOD, and GPx) and inflammatory responses (increased TNF-α, IL-1β, and IL-6 with reduced IL-10), contributing to granulosa cell apoptosis and follicular atresia. (**D**) Experimental evidence suggests that *Xylopia aethiopica* extract may partially ameliorate these alterations by improving hormonal profiles, ovarian morphology, and uterine histoarchitecture. This figure is intended as a conceptual integration of findings from multiple studies rather than a single experimental model.

These structural and functional alterations intensify with prolonged exposure and are consistent with progressive ovarian and reproductive tract remodeling. Mechanistically, AlCl_3_-associated female reproductive toxicity involves disruption of hypothalamic–pituitary–ovarian endocrine signaling, impaired steroidogenic enzyme activity, ovarian redox imbalance, mitochondrial and endoplasmic reticulum injury, altered gonadotropin receptor expression, and activation of apoptosis- and autophagy-related pathways, collectively contributing to defective follicular maturation, corpus luteum regression, and reproductive tissue degeneration, as illustrated in Figure 7.

## 8. Aluminum Chloride-Induced Thyroid and Multi-Axis Endocrine Disruption

Aluminum chloride exposure induces consistent thyroid dysfunction in experimental male rat models, primarily characterized by reductions in circulating triiodothyronine (T_3_) and thyroxine (T_4_), while pituitary responses (TSH) vary depending on exposure conditions. Almarzany (2020) reported decreased T_3_ and T_4_ without significant TSH alteration following oral exposure, suggesting impaired thyroid hormone output without overt pituitary compensation [32]. In contrast, Al Nahari and Al Eisa (2016) observed reductions in TSH, T_3_, T_4_, and the T_3_/T_4_ ratio after intraperitoneal administration, indicating suppression at both thyroidal and pituitary levels [33]. Conversely, Mekkey (2021) demonstrated dose-dependent decreases in T_3_ and T_4_ accompanied by elevated TSH at higher doses, consistent with a compensatory response to primary thyroid injury [101].

**Figure 8:**
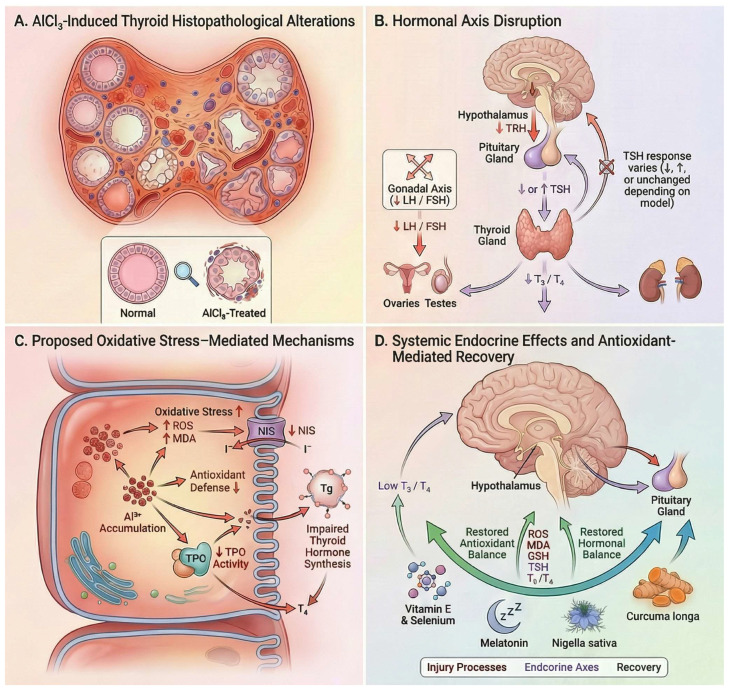
Conceptual overview of aluminum chloride (AlCl_3_)-induced thyroid and endocrine axis disruption in rats. (**A**) Experimentally observed thyroid histopathological alterations, including follicular epithelial degeneration, inflammatory infiltration, and reduced colloid content. (**B**) Disruption of hypothalamic–pituitary–thyroid and gonadal axes, characterized by decreased T_3_/T_4_ levels and variable TSH responses depending on exposure conditions, with reduced LH/FSH. (**C**) Proposed mechanisms, based on available evidence, involve oxidative stress-associated impairment of thyroid hormone synthesis and antioxidant defense. (**D**) Systemic endocrine effects and partial recovery following antioxidant or cytoprotective interventions (e.g., melatonin, *Nigella sativa*, *Curcuma longa*), associated with restoration of hormonal and redox balance.

Thus, decreased T_3_ and T_4_ represent the most consistent endocrine signature of AlCl_3_ toxicity, whereas TSH responses remain model-dependent (Figure 8). A plausible explanation is that aluminum exposure may disrupt thyroid hormone biosynthesis and secretion at the glandular level, while compensatory responses of the hypothalamic–pituitary–thyroid (HPT) axis vary according to exposure conditions [102]. Experimental evidence from aluminum exposure models indicates that aluminum can reduce thyroidal iodide uptake and iodide release, increase thyroid lipid peroxidation, and potentially impair sodium–iodide symporter-associated transport through oxidative membrane injury and Na^+^/K^+^-ATPase dysfunction [102].

Under some conditions, these alterations may remain partially compensated by the hypothalamic–pituitary–thyroid (HPT) axis, resulting in reduced T_3_/T_4_ without major TSH elevation [102]. Accordingly, variability in TSH responses across experimental studies may reflect differences in endocrine compensation dynamics and exposure-related conditions rather than contradictory evidence regarding thyroid toxicity.

Histopathological evidence supports direct thyroid injury as the underlying mechanism. Mekkey (2021) reported degeneration of follicular epithelial cells, inflammatory infiltration, necrotic changes, and depletion of follicular colloid, indicating disrupted follicular integrity and impaired hormone synthesis, which provides a structural basis for the observed hormonal decline [101].

Beyond the thyroid axis, AlCl_3_ induces broader endocrine disruption involving the pituitary–gonadal axis. Al Nahari and Al Eisa (2016) demonstrated significant reductions in FSH, LH, testosterone, and the T_3_/T_4_ ratio, together with marked testicular damage, including oligospermia, hypoplasia, and interstitial degeneration, indicating concurrent impairment of reproductive endocrine function [33].

Mechanistically, these effects are consistent with oxidative stress–mediated disruption of thyroid hormone synthesis and endocrine signaling. The partial reversal of hormonal and histological alterations by melatonin [32], *Nigella sativa* oil [101], and *Curcuma longa* [33] further supports the involvement of pathways responsive to antioxidant or cytoprotective modulation.

Overall, AlCl_3_ induces primary thyroid injury characterized by suppression of T_3_ and T_4_ and structural follicular damage, accompanied by variable hypothalamic–pituitary responses and multi-axis endocrine disruption affecting reproductive function.

## 9. Gastrointestinal Toxicity Induced by AlCl_3_: Molecular and Barrier-Level Mechanisms

**Figure 9:**
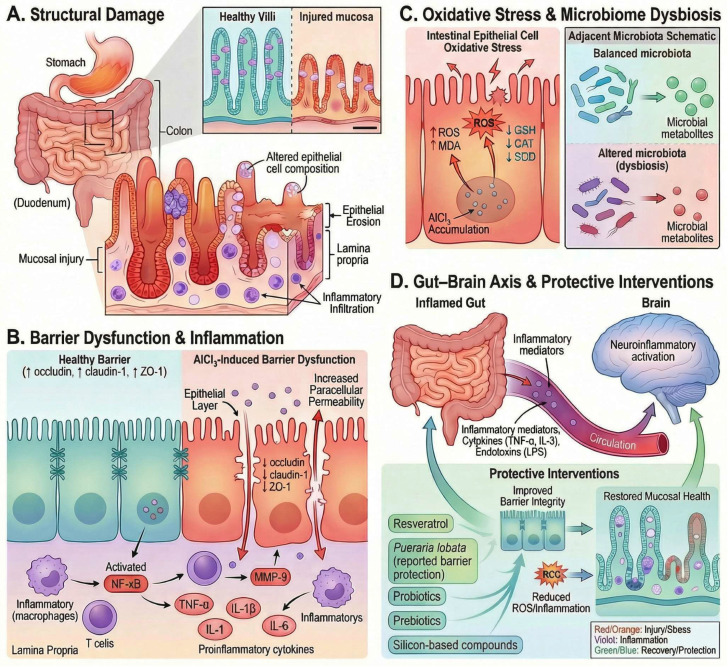
Conceptual overview of AlCl_3_-induced gastrointestinal toxicity and gut–brain axis interactions. Schematic representation of the proposed mechanisms underlying intestinal and systemic effects of chronic AlCl_3_ exposure. (**A**) Structural alterations of the intestinal mucosa, including epithelial erosion, altered cell composition, and inflammatory infiltration. (**B**) Disruption of epithelial barrier integrity characterized by reduced tight junction proteins (e.g., occludin, claudin-1, ZO-1), increased paracellular permeability, and activation of inflammatory signaling pathways (e.g., NF-κB, cytokine release). (**C**) Induction of oxidative stress in intestinal epithelial cells (increased ROS and lipid peroxidation, decreased antioxidant defenses) and associated shifts in gut microbiota composition (dysbiosis). (**D**) Potential systemic consequences via the gut–brain axis, including translocation of inflammatory mediators and endotoxins, contributing to neuroinflammatory responses, alongside illustrative protective interventions.

Chronic oral AlCl_3_ exposure induces gastrointestinal toxicity by disrupting oxidative and metabolic pathways and altering intestinal epithelial homeostasis, characterized by ROS accumulation, depletion of tight junction proteins (occludin, claudin-1, ZO-1), impaired epithelial barrier integrity, and increased paracellular permeability. These alterations are accompanied by NF-κB/ERK1/2-associated inflammatory activation, crypt and villous injury, metabolomic disturbances involving glutathione and mitochondrial energy metabolism, and microbiota dysbiosis, collectively promoting mucosal inflammation and systemic inflammatory signaling, as illustrated in Figure 9.

Direct evidence from intestinal epithelial models demonstrates that AlCl_3_ exposure markedly increases intracellular reactive oxygen species, including a substantial elevation in superoxide (~38-fold), while concurrently reducing the expression of tight junction proteins such as occludin and claudin-1 and significantly decreasing transepithelial electrical resistance, collectively indicating disruption of epithelial barrier integrity [34]. In the same study, AlCl_3_ exposure increased intestinal myeloperoxidase activity in vivo and activated ERK1/2 and NF-κB signaling, together with elevated TNF-α, IL-1β, and IL-6, indicating a coordinated oxidative and inflammatory response associated with epithelial barrier disruption [34].

This barrier dysfunction is further supported by inflammatory and junctional alterations in a murine model of chronic oral AlCl_3_ exposure. Hao et al. (2022) showed that AlCl_3_ induced intestinal pathological damage, including crypt abscesses, hyperplasia, villous shortening, and inflammatory cell infiltration, and increased intestinal barrier permeability, as evidenced by Evans blue extravasation and elevated serum DAO [35]. In the same model, AlCl_3_ increased intestinal IL-1β and IL-18, as well as serum IL-1β and TNF-α, while reducing the tight-junction proteins CLD1, OCLN, and ZO-1 at both mRNA and protein levels. These changes were accompanied by increased IRF8 and MMP9 expression, supporting the involvement of an IRF8–MMP9-associated mechanism in AlCl_3_-induced junctional disruption. Resveratrol partially reversed the histological injury, inflammatory response, permeability changes, tight-junction loss, and depressive-like behavior observed in AlCl_3_-exposed mice [35].

At the cellular level, AlCl_3_-induced epithelial injury extends beyond junctional disruption to broader metabolic and structural disturbances within enterocytes. In HT-29 cells exposed to 4 mM aluminum chloride, Yu et al. (2019) identified 81 significantly altered metabolites and 17 disrupted metabolic pathways, including glutathione metabolism, the tricarboxylic acid cycle, pyruvate metabolism, and multiple pathways related to lipid and amino-acid metabolism [103]. These changes included reduced glutathione, citrate, succinate, and several membrane-associated phospholipid species, together with altered expression of genes linked to redox control and mitochondrial metabolism, supporting a pattern of oxidative stress, impaired energy production, and membrane phospholipid disturbance. Consistent with these findings, the authors concluded that aluminum cytotoxicity in HT-29 cells involves cellular apoptosis, oxidative stress, and disruption of lipid, energy, and amino-acid metabolism [103].

In addition, AlCl_3_ exposure perturbs the intestinal microbiota, contributing to the dysbiotic state. In Wistar rats exposed to aluminum administered as AlCl_3_·6H_2_O, Wang et al. (2022) demonstrated a dose-dependent reduction in microbial diversity and a clear shift in overall community structure, as evidenced by 16S rRNA sequencing and multivariate analysis [104]. Aluminum exposure also altered the relative abundance of specific taxa, including reductions in beneficial genera such as Akkermansia and Dorea, alongside increases in potentially pathogenic bacteria such as Aggregatibacter, and was associated with changes in predicted microbial functional pathways. These findings support the presence of microbiota dysbiosis under AlCl_3_ exposure, which the authors identified as a potential mechanism contributing to aluminum toxicity. Although specific taxonomic alterations may vary across models, disruption of microbial homeostasis is mechanistically relevant, as it can indirectly influence intestinal barrier integrity and host inflammatory responses.

At the tissue level, AlCl_3_-induced gastrointestinal pathology is best characterized as mucosal injury accompanied by inflammatory activation. In murine models of oral AlCl_3_ exposure, Jeong et al. (2020) and Hao et al. (2022) reported intestinal pathological damage with histological features including crypt abscesses, villous shortening/blunting, epithelial injury, and inflammatory cell infiltration [34,35], while additional rat studies demonstrate colon inflammation and mucosal ulceration following oral AlCl_3_ administration [105]. However, specific morphometric features—such as villus atrophy, crypt hyperplasia, or defined goblet-cell alterations—are not consistently reported across AlCl_3_-models.

Finally, these intestinal alterations may have systemic consequences through the gut–brain axis. AlCl_3_-induced barrier dysfunction can facilitate the translocation of inflammatory mediators and endotoxin-derived signals into the circulation, contributing to systemic inflammation. Experimental studies in aluminum-exposed models have reported increased circulating pro-inflammatory cytokines, including TNF-α and IL-1β, consistent with this mechanism. While direct evidence linking intestinal permeability, endotoxemia, and microglial activation within a single AlCl_3_-only model remains limited, available findings support the occurrence of systemic and neuroinflammatory responses downstream of intestinal dysfunction, consistent with the pathway illustrated in Panel D.

Collectively, these findings support a mechanistic model in which AlCl_3_ accumulates in the intestinal environment, inducing oxidative stress, disrupting tight junction integrity, and activating inflammatory signaling pathways (e.g., NF-κB). Concurrently, metabolomic alterations and lipid dysregulation contribute to metabolic and membrane instability in enterocytes, while shifts in gut microbial composition reflect microbiota dysbiosis. Together, these processes converge to promote intestinal barrier dysfunction and mucosal inflammation, establishing the gastrointestinal tract as both a primary target and a critical mediator of systemic toxicity following AlCl_3_ exposure.

## 10. Molecular and Cellular Mechanisms of Aluminum Chloride–Induced Pancreatic Dysfunction and Glucose Homeostasis Disruption

Chronic AlCl_3_ exposure induces metabolic dysfunction characterized by impaired glucose homeostasis. Experimental studies demonstrate that prolonged oral AlCl_3_ administration results in significant fasting hyperglycaemia and systemic metabolic disturbance, reflecting disruption of glucose regulatory mechanisms. In a chronic rat model, prolonged AlCl_3_ exposure induced hyperglycemia together with depletion of glutathione in the hippocampus and frontal cortex, supporting disruption of systemic metabolic homeostasis accompanied by oxidative injury within insulin-sensitive neural regions rather than evidence of an isolated pancreatic lesion [106]. In parallel, pancreatic-focused studies report structural alterations within the endocrine pancreas, including a significant reduction in islet cell number and histopathological evidence of coagulative necrosis and architectural disorganization of pancreatic islets following chronic oral AlCl_3_ exposure, supporting the presence of direct β-cell injury as a likely contributor to impaired insulin production and disrupted glycaemic regulation [36].

These structural abnormalities are accompanied by dynamic metabolic disturbances rather than a uniform functional decline. Experimental evidence demonstrates time-dependent alterations in circulating insulin levels, characterized by an initial increase followed by a subsequent reduction with prolonged exposure, together with an early elevation in insulin resistance indices (HOMA-IR) and a consistent decrease in β-cell functional capacity (HOMA-β), reflecting progressive β-cell dysfunction [107]. This evolving metabolic profile indicates that AlCl_3_-induced pancreatic injury is associated with an initial compensatory phase of insulin dysregulation that transitions toward impaired endocrine function and systemic disturbance of glucose homeostasis rather than a single static metabolic state.

At the molecular level, chronic AlCl_3_ exposure disrupts redox-dependent neuronal homeostasis within the hippocampus and frontal cortex, characterized by marked depletion of reduced glutathione (GSH), impaired antioxidant buffering capacity, and oxidative modification of cholinergic regulatory systems in brain regions critically involved in spatial learning and memory [106]. This oxidative disruption is accompanied by metabolic interference, including inhibition of key enzymes involved in glucose utilization (e.g., hexokinase and glucose-6-phosphate dehydrogenase), which contributes to impaired glucose homeostasis and hyperglycaemia observed in experimental models [106]. Together, these findings support a mechanism in which oxidative stress and metabolic enzyme dysfunction act in concert to compromise cellular viability and systemic metabolic regulation. However, while oxidative stress–mediated injury and metabolic disruption are well supported, direct evidence linking AlCl_3_ exposure to specific intracellular insulin signaling defects (e.g., IRS-1 or Akt dysregulation) within pancreatic or peripheral tissues remains limited in AlCl_3_-specific models.

Beyond pancreatic injury, AlCl_3_ exposure is associated with broader metabolic and neurobiological disturbances. In a chronic oral rat model, prolonged AlCl_3_ administration produced significant hyperglycaemia together with marked depletion of glutathione and reduced acetylcholinesterase activity in the hippocampus and frontal cortex, in parallel with impaired spatial memory, indicating that systemic metabolic disturbance can coexist with oxidative and cholinergic dysfunction in cognition-related brain regions [106]. Additional support for this metabolic–neurodegenerative convergence comes from a diabetes-associated Alzheimer-like model in which AlCl_3_ was superimposed on nicotinamide/streptozotocin-induced type 2 diabetes; in that setting, diseased animals exhibited elevated blood glucose, increased lipid peroxidation, reduced antioxidant defenses, hippocampal plaque deposition, and neuronal degeneration, all of which were attenuated by dulaglutide treatment [108]. Accordingly, these findings suggest that AlCl_3_ can participate in a broader neuro-metabolic injury pattern characterized by disturbed glucose regulation, oxidative stress, and cognitive impairment, while diabetes-associated conditions may further amplify Alzheimer-like pathology. The broader “type 3 diabetes” framework provides a useful conceptual context for interpreting these overlaps, as accumulating evidence links impaired insulin signaling and disrupted cerebral glucose metabolism with defective PI3K/Akt pathway activity, reduced neuronal glucose uptake, altered amyloid-β clearance, tau hyperphosphorylation, mitochondrial bioenergetic failure, neuroinflammatory activation, and progressive cognitive dysfunction characteristic of Alzheimer-related neurodegeneration [109]. Within the scope of the present review, this framework is best used to suggest that AlCl_3_-induced metabolic dysregulation may intersect with pathways commonly discussed in the type 3 diabetes literature, rather than to imply that AlCl_3_ toxicity itself establishes a formally recognized type 3 diabetes model or a direct pancreas-to-brain causal axis. Atabi et al. likewise emphasize that the term remains conceptual and debated, is not formally recognized by major health organizations, and should therefore be applied cautiously when extrapolating from reductionist experimental systems such as AlCl_3_ exposure.

Intervention studies further support a contributory role of oxidative stress and metabolic dysregulation in AlCl_3_-based models, while also highlighting the importance of impaired central insulin signaling. Specifically, pharmacological interventions such as GLP-1 receptor agonists (e.g., dulaglutide) and standard cholinesterase inhibitors (e.g., donepezil) have demonstrated therapeutic benefits. Dulaglutide, in particular, significantly reduced blood glucose levels, decreased lipid peroxidation, and restored antioxidant defenses (SOD, GSH, catalase), while also improving acetylcholine levels and reducing acetylcholinesterase activity. These biochemical and neurochemical improvements were accompanied by reduced neuronal degeneration and amyloid plaque burden, as well as enhanced cognitive performance in AlCl_3_-induced diabetic Alzheimer’s models [108]. Mechanistically, the reported effects are linked to modulation of interconnected pathways shared by metabolic and neurodegenerative disorders, including attenuation of oxidative stress, improvement of mitochondrial function, promotion of autophagy-related signaling, and regulation of PI3K/Akt/mTOR and Wnt/β-catenin pathways, thereby potentially supporting neuronal survival, synaptic plasticity, and cognitive performance [110]. Additionally, in an AlCl3-induced Alzheimer-like rat model, *Peganum harmala* was shown to improve hippocampal insulin-signaling markers, as reflected by reduced inhibitory IRS-1 Ser307 phosphorylation, increased Akt Ser473 phosphorylation, and elevated GLUT4 content, alongside higher hippocampal insulin and GLP-1 levels. These changes were accompanied by reductions in Aβ42 and phosphorylated tau, as well as improved oxidative-stress markers, supporting a hippocampal insulin-sensitizing and antioxidant-associated mechanism rather than proving direct restoration of brain insulin signaling in humans.

Overall, available evidence suggests that chronic AlCl_3_ exposure is associated with oxidative stress–related metabolic disturbances affecting both pancreatic and neural systems. These changes may contribute to impaired insulin regulation, disrupted glucose homeostasis, and, in the brain, alterations linked to reduced glucose utilization and accumulation of markers such as amyloid-β and phosphorylated tau, in line with mechanisms discussed in the “type 3 diabetes” framework.

**Figure 10:**
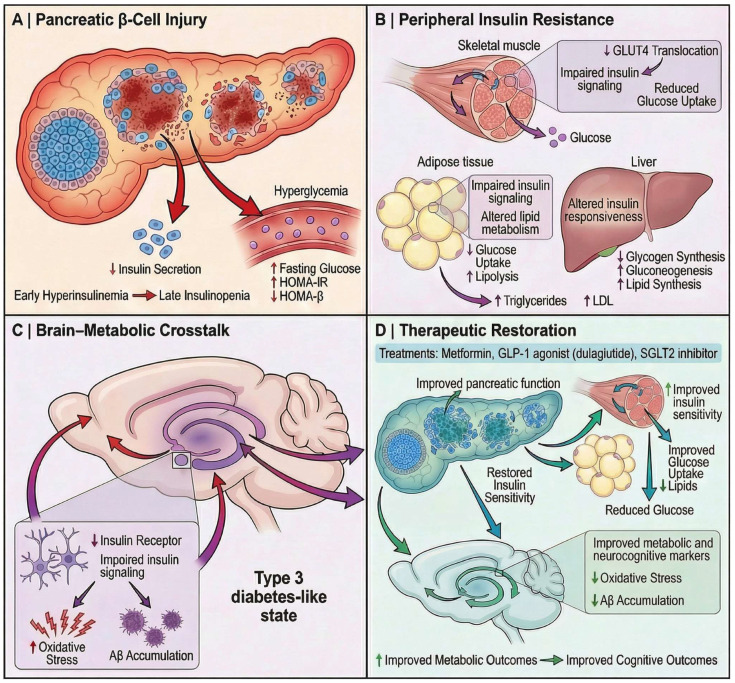
Conceptual overview of potential pancreatic, peripheral, and central alterations associated with AlCl_3_ exposure. (**A**) Proposed features of pancreatic β-cell injury and altered insulin dynamics. (**B**) Schematic representation of peripheral insulin resistance across muscle, adipose tissue, and liver. (**C**) Putative brain–metabolic interactions involving impaired insulin signaling, oxidative stress, and neurodegeneration-related changes. (**D**) Illustrative summary of reported effects of pharmacological interventions on metabolic and neurocognitive parameters.

Pharmacological interventions appear to partially improve these alterations through antioxidant and metabolic pathway modulation; however, the relative contributions of central versus peripheral effects, as well as pancreas–brain interactions, remain unclear. Accordingly, Figure 10 represents an integrative conceptual framework grounded in current evidence, rather than a singular validated pathway.

## 11. Musculoskeletal Toxicity in Chronic Aluminum Chloride Exposure

Chronic exposure to AlCl_3_ extends systemic toxicity to the musculoskeletal system, although the depth of experimental evidence differs between skeletal muscle and bone. In skeletal muscle, current data primarily support metabolic and functional disturbances rather than well-characterized structural pathology. In particular, AlCl_3_ exposure in rats has been shown to disrupt glucose homeostasis, as evidenced by increased fasting blood glucose levels and elevated insulin resistance indices (HOMA-IR), particularly during the early phase of exposure, together with time-dependent alterations in circulating insulin levels and progressive impairment of pancreatic structure. These metabolic disturbances are accompanied by a significant reduction in glucose transporter 4 (GLUT4) mRNA and protein expression in skeletal muscle, indicating impaired insulin-mediated glucose uptake and contributing to peripheral metabolic dysfunction [107].

Mechanistic insight into skeletal muscle involvement is supported by experimental studies in isolated muscle preparations demonstrating that AlCl_3_ exerts concentration-dependent effects on calcium-related processes. Specifically, increasing concentrations of AlCl_3_ have been shown to induce a progressive reduction in sarcoplasmic reticulum Ca^2+^,Mg^2+^-ATPase activity, accompanied by a corresponding decrease in contractile force and muscle fiber shortening, with complete suppression of contraction observed at higher concentrations. These findings suggest that aluminum may disrupt intracellular calcium regulation and impair contractile performance under experimental conditions [37].

Collectively, these findings support a model in which AlCl_3_-induced skeletal muscle toxicity is primarily mediated through metabolic dysregulation and impaired calcium handling, although direct in vivo histopathological characterization remains limited.

Collectively, these findings support a model in which AlCl_3_-induced skeletal muscle alterations are primarily associated with metabolic dysregulation and impaired calcium handling, although direct in vivo histopathological characterization remains limited. In contrast, bone tissue represents a major target of aluminum toxicity, where the primary pathogenic mechanism is inhibition of bone formation mediated by osteoblast dysfunction [38]. Experimental evidence demonstrates that AlCl_3_ exposure significantly reduces osteoblast viability and suppresses the expression of key osteogenic growth-regulatory factors, including TGF-β1, BMP-2, IGF-I, and Cbfα1, which are essential for osteoblast proliferation and differentiation [38]. In parallel, AlCl_3_ induces oxidative stress in osteoblasts, as reflected by increased reactive oxygen species levels and decreased antioxidant enzyme activities, including superoxide dismutase and glutathione peroxidase. These biochemical alterations are accompanied by pronounced ultrastructural damage, including mitochondrial swelling, nuclear membrane disruption, and cytoplasmic disorganization, collectively indicating severe impairment of osteoblast function and viability [38].

These alterations are mechanistically associated with inactivation of the Wnt/β-catenin signaling pathway, a key regulator of osteoblast differentiation and bone formation. In vivo studies in rats demonstrate that AlCl_3_ exposure suppresses this pathway through upregulation of Wnt antagonists, including Dkk1 and sFRP1, reduction in the p-GSK3β/GSK3β ratio, and decreased β-catenin expression, ultimately leading to downregulation of osteogenic markers such as type I collagen and IGF-1 and consequent inhibition of bone formation [111].

At the cellular level, AlCl_3_ directly impairs osteoblast function, as demonstrated in primary rat osteoblast cultures where AlCl_3_ exposure suppresses osteoblastic differentiation, evidenced by reduced alkaline phosphatase activity and downregulation of osteogenic markers such as type I collagen and Runx2. These effects are accompanied by inactivation of the canonical Wnt/β-catenin signaling pathway, reflected by decreased β-catenin stabilization and nuclear translocation, reduced p-GSK3β/GSK3β ratio, downregulation of Wnt3a, and upregulation of the antagonist Dkk1. Importantly, exogenous Wnt3a reverses these inhibitory effects, confirming that suppression of osteoblastic differentiation by AlCl_3_ is mechanistically mediated through Wnt/β-catenin pathway inactivation [112]. In addition, AlCl_3_ induces oxidative stress–mediated apoptosis in osteoblasts, as demonstrated by increased reactive oxygen species generation and reduced antioxidant enzyme activity, accompanied by activation of the c-Jun N-terminal kinase signaling pathway, including increased JNK phosphorylation and upregulation of c-Jun expression. This activation is associated with elevated expression of pro-apoptotic genes such as caspase-3, caspase-9, bax, and FASL, together with downregulation of the anti-apoptotic protein Bcl-2, collectively leading to increased osteoblast apoptosis and reduced osteogenic function [113].

Complementary evidence further demonstrates that AlCl_3_ disrupts intracellular calcium homeostasis in osteoblasts, as reflected by increased intracellular Ca^2+^ concentration and altered calmodulin expression, leading to activation of the Ca^2+^/CaMKII signaling pathway, evidenced by elevated phosphorylation of CaMKII. This pathway activation is directly linked to increased osteoblast apoptosis, and notably, pharmacological chelation of intracellular Ca^2+^ using BAPTA-AM attenuates these effects, confirming a causal role of calcium dysregulation in AlCl_3_-induced osteoblast injury [114].

Consistent with these mechanisms, AlCl_3_ has been shown to suppress osteoblast function in vitro, as evidenced by a dose-dependent reduction in cell viability, accompanied by downregulation of key osteogenic growth-regulatory genes, including TGF-β1, BMP-2, IGF-I, and Cbfα1. These effects are further associated with impaired antioxidant capacity, reflected by decreased superoxide dismutase and glutathione peroxidase activities and increased reactive oxygen species levels, together with pronounced ultrastructural alterations such as mitochondrial swelling and membrane disruption, collectively indicating direct functional and structural impairment of osteoblasts [115].

Importantly, intervention-based studies provide functional support for this pathological framework. In a rat model of AlCl_3_-induced bone impairment, administration of ginsenoside Rg3 attenuated structural damage to the femur, improved osteoblast activity, differentiation, and mineralization, and reduced apoptosis in both bone tissue and osteoblastic cells. These protective effects were accompanied by restoration of extracellular matrix-related gene expression and reactivation of the TGF-β1/Smad signaling pathway, with pathway inhibition experiments further confirming its mechanistic involvement, indicating that AlCl_3_-induced skeletal alterations are, at least in part, reversible under targeted intervention [116].

**Figure 11:**
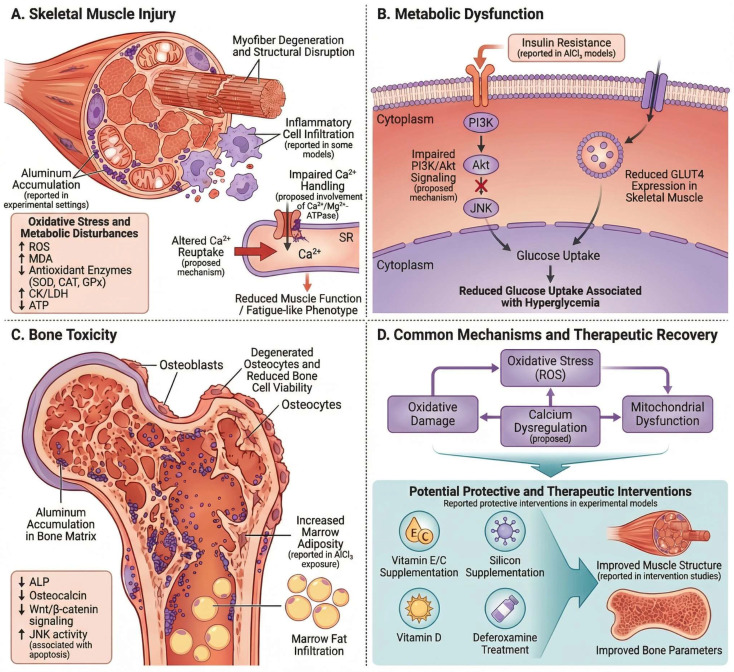
Conceptual overview of musculoskeletal and metabolic alterations associated with chronic aluminum chloride (AlCl_3_) exposure. This schematic represents an integrative, hypothesis-driven summary of experimentally reported findings rather than a single validated pathway. (**A**) Skeletal muscle alterations include oxidative stress, mitochondrial dysfunction, inflammatory responses, and impaired Ca^2+^ handling, which may contribute to reduced contractile function and fatigue-like phenotypes. (**B**) Metabolic dysfunction is characterized by impaired PI3K/Akt signaling, reduced GLUT4 expression, and decreased glucose uptake, consistent with insulin resistance and hyperglycemia observed in experimental models. (**C**) Bone toxicity involves osteoblast dysfunction, oxidative stress, and suppression of Wnt/β-catenin signaling, leading to reduced bone formation, osteocyte degeneration, and increased marrow adiposity. (**D**) Shared mechanisms across tissues include oxidative stress, calcium dysregulation, and mitochondrial impairment. Potential protective interventions (e.g., antioxidants, silicon compounds, vitamin D, and chelation therapy) are shown as reported in experimental studies.

Taken together, the available evidence supports the interpretation that chronic AlCl_3_ exposure produces a direct toxic osteopathy characterized by impaired osteoblast function, suppression of Wnt/β-catenin signaling, oxidative stress–driven apoptosis, and disruption of calcium homeostasis, ultimately leading to reduced bone formation and progressive deterioration of skeletal integrity, as shown in Figure 11.

## 12. Integrated Molecular Architecture of Systemic AlCl_3_ Toxicity

Importantly, the integrative framework proposed in this review is intended to organize experimentally observed toxicological responses to chronic AlCl_3_ exposure across organ systems and should not be interpreted as evidence that aluminum exposure fully recapitulates the clinical or etiological spectrum of Alzheimer’s disease.

**Figure 12:**
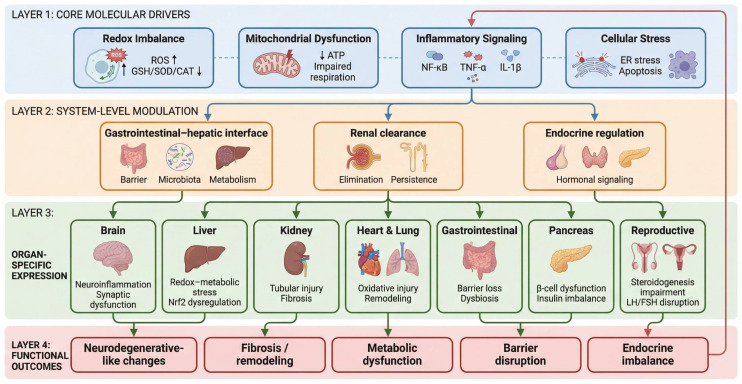
Conceptual multi-layer framework of systemic aluminum chloride (AlCl_3_) toxicity. This schematic illustrates an integrative, systems-level model derived from experimental evidence and is intended as a conceptual synthesis rather than a single validated pathway. The framework comprises four interconnected layers: (1) core molecular drivers, including oxidative stress, mitochondrial dysfunction, inflammatory signaling, and cellular stress responses; (2) system-level modulation through the gastrointestinal–hepatic interface, renal clearance, and endocrine regulation; (3) organ-specific expression across major tissues (brain, liver, kidney, heart and lung, gastrointestinal tract, pancreas, and reproductive organs); and (4) functional outcomes, including neurodegenerative-like changes, fibrosis, metabolic dysfunction, barrier disruption, and endocrine imbalance. Arrows represent proposed interactions and do not imply direct causality.

When considered collectively, the available evidence instead supports a conceptual interpretation of chronic AlCl_3_ toxicity as a distributed molecular stress state characterized by recurrent and interacting cellular responses across tissues rather than isolated organ-specific lesions. As schematically illustrated in Figure 12, this framework can be resolved into three interconnected levels comprising shared molecular drivers, system-level modulation, and tissue-specific expression.

At the molecular level, the most consistently observed cross-organ alteration involves disruption of cellular redox buffering capacity, characterized by excessive lipid peroxidation, hydrogen peroxide accumulation, and progressive depletion of endogenous antioxidant systems including glutathione (GSH), superoxide dismutase (SOD), and catalase (CAT), collectively promoting membrane destabilization, enzyme dysfunction, and impaired cellular metabolic homeostasis across affected tissues [7,8]. This redox imbalance is closely coupled to mitochondrial dysfunction, including impaired respiratory chain activity and reduced ATP production, indicating that disrupted bioenergetic homeostasis represents a parallel and interacting component of the injury profile rather than a purely downstream consequence [56,59]. In parallel, inflammatory activation recurs across tissues, most commonly involving NF-κB-associated signaling and increased expression of cytokines such as TNF-α, IL-1β, and IL-6 [7,60]. These processes converge on cellular stress responses, including apoptosis mediated by caspase-3 activation and Bax/Bcl-2 imbalance [11,19], while in selected tissues additional modules—such as endoplasmic reticulum stress signaling (BiP/GRP78–CHOP–XBP1) [64] and kinase-associated pathways, including GSK3β [11], further refine local injury patterns. Within this framework, these pathways are best interpreted as interacting molecular modules whose relative contribution varies across tissues rather than as components of a single linear cascade.

A second level of integration arises from organ systems that modulate the systemic expression of toxicity through their roles in exposure handling and internal regulation, as highlighted in Figure 12. The GIT and liver function as primary interfaces of exposure and metabolic processing, where epithelial barrier disruption [34,35], characterized by reduced expression of tight junction proteins such as occludin, claudin-1, and ZO-1, together with microbiota dysbiosis [104] and hepatocellular redox–metabolic stress, including dysregulation of the Nrf2 pathway [19,42,64,69], may shape the systemic inflammatory and oxidative milieu. The kidney introduces an additional regulatory dimension through its role in elimination, such as tubular oxidative injury, activation of pro-inflammatory mediators, and fibrogenic signaling pathways such as TGF-β/Smad may prolong internal exposure and reinforce molecular stress [22,24]. Endocrine disruption further extends this integrative layer by altering hormonal signaling pathways, including reductions in T_3_ and T_4_, disturbances in LH, FSH, testosterone, estradiol, and progesterone [32,33,101], and dysregulation of insulin signaling [106,107], thereby influencing tissue responsiveness across multiple organ systems.

Within this shared molecular and systemic context, organ-specific phenotypes can be interpreted as tissue-dependent expressions of a common disturbance. In the central nervous system, the redox–inflammatory core is associated with synaptic dysfunction, reduced BDNF and synaptophysin, increased acetylcholinesterase activity, and partial amyloid-β accumulation with GSK3β-related tau phosphorylation [7,11]. In hepatic [19,42] and renal tissues [22,24], similar upstream processes are coupled to metabolic disruption, ER stress, apoptosis, and progressive structural remodeling, with renal injury exhibiting a more pronounced fibrogenic component and hepatic injury more closely linked to redox-regulatory pathways such as Nrf2 signaling. In cardiac [25,77,78] and pulmonary tissues [81,82]. These mechanisms manifest as oxidative–inflammatory injury, membrane destabilization, cytokine-associated structural alterations, and early extracellular matrix remodeling. In reproductive and endocrine systems, the same underlying disturbance is expressed through impaired steroidogenesis, disrupted gonadotropin signaling, mitochondrial dysfunction, and activation of apoptotic and autophagic pathways affecting germinal and follicular cells [28,89,90,91]. In metabolic tissues, including the pancreas, these processes are associated with β-cell injury, altered insulin dynamics, increased insulin resistance indices, and impaired glucose utilization [36,106,107]. These differences are most consistently explained by variation in cellular composition, metabolic demand, and physiological function rather than by distinct initiating mechanisms.

Taken together, the available evidence supports a conceptual systems-level interpretation of aluminum-induced toxicity comprising three interacting dimensions: recurrent molecular stress modules shared across tissues, organ-specific expression shaped by local physiological context, and systemic modulation influenced by exposure handling, clearance, barrier integrity, and endocrine regulation. This framework does not imply that all inter-organ interactions have been causally established but rather provides a structured basis for integrating heterogeneous findings and identifying common mechanistic themes. In this sense, the value of the AlCl_3_ model lies in its ability to illustrate how a limited set of molecular disturbances can give rise to diverse yet mechanistically related pathological outcomes across biological systems.

## 13. Clinical Relevance and Human Exposure Considerations

Although experimental AlCl_3_ models provide valuable mechanistic insight into aluminum-associated toxicity, their translational interpretation requires caution. Human exposure is usually chronic, low-level, and heterogeneous, arising mainly from food, drinking water, food-contact materials, pharmaceuticals, cosmetics, and occupational inhalation rather than from the high-dose controlled paradigms commonly used in animal studies. Recent food-exposure assessments continue to identify dietary intake as a relevant exposure route, while JECFA maintains a provisional tolerable weekly intake of 2 mg/kg body weight for aluminum compounds in food [117,118].

A central translational limitation is toxicokinetics. Oral aluminum absorption is generally low and varies with physicochemical form, gastrointestinal conditions, age, and renal clearance capacity, underscoring the importance of exposure route and toxicokinetic context when interpreting experimental findings [119].

Clinical relevance is strongest in contexts of elevated exposure or impaired elimination. Aluminum toxicity is now uncommon in modern dialysis practice but remains clinically recognized in renal failure or dialysis-related accumulation, where encephalopathy, osteomalacia/osteoporosis, and anemia may occur. Occupational evidence also remains relevant; a 2024 meta-analysis reported poorer processing speed, working memory, attention, and reaction time among aluminum-exposed workers, with plasma aluminum identified as a significant predictor of cognitive performance [120].

Regarding Alzheimer’s disease, recent evidence still supports caution rather than causation. A 2025 systematic review and meta-analysis evaluated environmental aluminum exposure in relation to AD risk, while recent reviews emphasize that aluminum may have neurotoxic potential under conditions of overexposure, but systematic evidence supporting a causal role in human neurodegenerative disease remains insufficient [121,122].

Accordingly, the main translational value of chronic AlCl_3_ models lies not in directly reproducing typical human exposure or proving disease causation, but in identifying conserved toxicological pathways that may become clinically relevant under specific vulnerability conditions, including occupational burden, impaired renal clearance, prolonged medical exposure, aging-related susceptibility, or compromised epithelial and blood–brain barrier function. These models are therefore best interpreted as mechanistic tools for understanding aluminum-associated stress responses rather than direct simulations of ordinary human exposure.

## 14. Conclusions

Current understanding of aluminum chloride (AlCl_3_) toxicity remains conceptually fragmented, as most studies have focused on individual organs—particularly the central nervous system—without adequately integrating findings across biological systems. This limitation has constrained the interpretation of aluminum toxicity as a coordinated, system-wide process driven by shared molecular disturbances.

This review addresses this gap by synthesizing evidence across multiple organ systems and proposing a unified molecular framework that organizes aluminum-induced effects into interconnected levels spanning molecular disruption, systemic modulation, and tissue-specific expression. Within this perspective, AlCl_3_ toxicity is more appropriately understood as a distributed biological process, in which common molecular stress responses are differentially expressed across organs depending on physiological context, rather than as a series of independent pathological events.

The primary contribution of this work lies in reframing the experimental use of AlCl_3_ beyond its conventional application in AD-like and neurotoxicity induction paradigms toward a broader interpretation as a systemic, molecularly driven toxicological exposure model. Importantly, the reviewed studies should not be interpreted as direct evidence that aluminum constitutes a definitive primary etiological cause of human neurodegenerative disease, but rather as controlled experimental systems used to reproduce selected neurodegenerative and organ-specific pathological features under defined exposure conditions. By integrating heterogeneous findings into a coherent structure, this framework provides a foundation for interpreting variability across studies and identifying shared mechanistic patterns across biological systems.

Although conceptual, this systems-oriented molecular perspective offers a basis for future research aimed at clarifying inter-organ interactions, improving experimental design, and guiding the development of more integrative toxicological and therapeutic strategies.

## Figures and Tables

**Table 1:** Representative experimental AlCl_3_ exposure paradigms across organ systems, summarizing biological models, exposure conditions, and principal pathological outcomes. The studies illustrate the use of AlCl_3_ primarily as a controlled toxicological induction.

Organ/System	Experimental Context	Species/Strain	Sample Size (n)	Dose Regimen	Route of Administration	Exposure Duration	Main Outcomes/Phenotype	Reference
Central nervous system	AD-like neurodegeneration induction model	Male Wistar rats	6/group (30 total)	100 mg/kg AlCl_3_	Oral gavage	42 days	Cognitive impairment with cholinergic dysfunction and redox imbalance	[7]
Central nervous system	AD-like neurodegeneration induction model	Male Wistar rats	6/group (24 total)	100 mg/kg AlCl_3_	Oral gavage	14 days	Cognitive deficits with cerebellar neurodegeneration and neuroinflammatory alterations	[8]
Central nervous system	AD-like neurodegeneration induction model	Female Sprague–Dawley rats	8/group (40 total)	100 mg/kg AlCl_3_	Oral gavage	8 weeks	Cognitive impairment with β-amyloid accumulation, Tau elevation, and apoptotic activation	[11]
Central nervous system	AD-like neurotoxicity induction model	Young adult male Wistar rats	6/group (24 total)	150, 300, or 600 mg/kg AlCl_3_	Oral gavage	8 weeks	Spatial memory impairment without detectable hippocampal senile plaque formation	[14]
Central nervous system	AD-like neurotoxicity induction model	Male Wistar rats	6/group (30 total)	1.5, 8.3, or 100 mg/kg/day AlCl_3_	Drinking water (1.5 and 8.3 mg/kg/day) or oral gavage (100 mg/kg/day)	60 days (1.5 and 8.3 mg/kg/day) or 42 days (100 mg/kg/day)	Recognition memory impairment with elevated hippocampal AChE activity and lipid peroxidation	[15]
Central nervous system	AD-like neurotoxicity induction model	Male Wistar rats	12/group (24 total)	8.3 mg/kg/day AlCl_3_	Oral gavage	60 days	Spatial learning deficits with hippocampal neuronal loss and proteomic remodeling	[16]
Central nervous system	Developmental neurotoxicity induction model	Male Wistar rats	7–9/group	0.2%, 0.4%, or 0.6% AlCl_3_	Drinking water exposure	3 months	Spatial memory impairment with synaptic ultrastructural disruption and impaired hippocampal L-LTP	[17]
Central nervous system	AD-like neurodegeneration induction model	Male Swiss Albino Wistar rats	10/group (40 total)	100 mg/kg AlCl_3_	Oral gavage	60 days	Cognitive impairment with β-amyloid deposition, neurofibrillary pathology, and elevated AChE activity	[18]
Hepatorenal system	AlCl_3_-induced hepatorenal toxicity model	Male Sprague–Dawley rats	6/group (24 total)	40 mg/kg AlCl_3_	Oral administration	2 months	Hepatorenal injury with Nrf2 suppression, apoptotic activation, and tissue architectural degeneration	[19]
Hepatorenal system	AlCl_3_-induced hepatorenal histopathological toxicity model	Male Wistar rats	8/group (32 total)	128 mg/kg AlCl_3_	Oral gavage	12 weeks	Hepatic necro-inflammatory injury with hepatocyte ballooning, sinusoidal congestion, and renal inflammatory infiltration	[20]
Multi-organ system (brain, liver, kidney)	AlCl_3_-induced multi-organ toxicity model	Adult albino rats	6/group (36 total)	20 mg/kg AlCl_3_	Intraperitoneal injection	60 days	Cortical neurodegeneration with hepatic vacuolation, renal tubular injury, and elevated hepatic/renal injury markers	[21]
Renal system	AlCl_3_-induced nephrotoxicity model	Male Wistar rats	6/group (24 total)	10 mg/kg AlCl_3_	Intraperitoneal injection	5 weeks	Renal fibrosis and tubular degeneration associated with MMP-9 upregulation and podocyte injury	[22]
Hepatorenal system	Chronic AlCl_3_ hepatorenal toxicity study	Male Wistar rats	30/group (90 total)	100 or 200 mg/kg AlCl_3_	Oral administration	30, 60, or 90 days	Progressive hepatic and renal aluminum accumulation with glomerular collapse, tubular hyperplasia, hepatic necrosis, and periportal fibrosis	[23]
Renal system	AlCl_3_-induced nephrotoxicity model	Male Wistar rats	8/group (32 total)	5, 10, or 20 mg/kg/day AlCl_3_	Intraperitoneal injection	4 weeks	Renal tubular injury with Kim-1 elevation, collagen deposition, and TGF-β1/Smad2-associated fibrosis	[24]
Neurocardiovascular system	AlCl_3_-induced neurocardiac oxidative stress study	Adult male Wistar rats	8/group (32 total)	100 mg/kg/day AlCl_3_	Oral administration	30 days	Neurocardiac injury with dyslipidemia, cholinergic suppression, nitric oxide depletion, and histological degeneration	[25]
Respiratory system	AlCl_3_-induced pulmonary oxidative stress study	Female Wistar rats	6/group (24 total)	50 mg/kg bw AlCl_3_	Drinking water exposure	21 days	Pulmonary injury with alveolar edema, emphysema, hemosiderin-laden macrophages, and altered pulmonary LDH activity	[26]
Respiratory system	AlCl_3_-induced pulmonary histopathological toxicity model	Male Sprague–Dawley albino rats	10/group (30 total)	475 mg/kg bw AlCl_3_	Oral gavage	8 weeks	Diffuse pulmonary architectural damage with alveolar collapse, septal thickening, hemorrhage, and mitochondrial degeneration	[27]
Reproductive system	AlCl_3_-induced testicular toxicity study	Male rats	6/group (24 total)	64.18, 128.36, or 256.72 mg/kg/day AlCl_3_	Drinking water exposure	16 weeks	Testicular degeneration with impaired spermatogenesis, steroid hormone suppression, and sperm-associated proteomic dysregulation	[28]
Reproductive system	AlCl_3_-induced reproductive toxicity model	Adult male albino rats	10/group (60 total)	100 mg/kg AlCl_3_	Oral gavage	5 weeks	Testicular dysfunction with impaired fertility indices, steroidogenic gene suppression, and Leydig cell degeneration	[29]
Reproductive system (ovary)	AlCl_3_-induced ovarian toxicity and granulosa cell dysregulation model	Immature female NMRI mice	5/group (20 total)	1.2, 4.8, or 12.1 mg/kg AlCl_3_	Intraperitoneal injection	Single administration with 2-week follow-up	Disrupted folliculogenesis with granulosa cell apoptosis and granulosa cell tumor-like ovarian alterations	[30]
Reproductive system (female)	AlCl_3_-induced female reproductive toxicity study	Female albino mice	18/group in toxicity experiments (36 total per experiment)	221.83 mg/kg (subacute) or 55.45 mg/kg (subchronic) AlCl_3_	Intraperitoneal injection	14 days (subacute) or 60 days (subchronic)	Ovarian, oviductal, and uterine degeneration with papillary endometrial hyperplasia and progressive systemic toxicity	[31]
Hematological/endocrine system	AlCl_3_-induced hematological and thyroid dysfunction study	Adult male albino rats	5/group (15 total)	1000 mg/L AlCl_3_	Drinking water exposure	40 days	Thyroid hormone dysregulation with hematological alterations and elevated serum/brain β-amyloid levels	[32]
Endocrine and reproductive system	AlCl_3_-induced pituitary–thyroid–testicular dysfunction model	Adult albino rats	6/group (36 total)	30 mg/kg AlCl_3_ every other day	Intraperitoneal injection	8 weeks	Pituitary–thyroid–testicular dysfunction with oligospermia, seminiferous tubular hypoplasia, and Leydig cell degeneration	[33]
Gastrointestinal system (intestinal epithelium/colon)	AlCl_3_-induced intestinal epithelial barrier dysfunction and colonic inflammation model	Human HT-29 colorectal epithelial cells and male C57BL/6 mice	HT-29 cells: independent in vitro experiments (typically n = 3 wells/group); mice: 8/group (32 total)	HT-29 cells: 1–4 mM AlCl_3_ (up to 24 h); mice: 5, 25, or 50 mg/kg BW AlCl_3_	HT-29 cells: direct culture exposure; mice: oral gavage	HT-29 cells: 1–24 h; mice: 13 weeks (5 d/week)	Intestinal barrier dysfunction with tight-junction disruption, crypt abscesses, villous blunting, and colonic inflammation	[34]
Gastrointestinal system (intestinal barrier/colon)	Subchronic AlCl_3_-induced intestinal barrier dysfunction model	SPF Kunming mice	10/group (50 total)	30.3, 101, or 303 mg/kg AlCl_3_; ± 100 mg/kg resveratrol	Oral administration (AlCl_3_); oral gavage (resveratrol)	3 months	Intestinal permeability dysfunction with crypt abscesses, villous shortening, IRF8-MMP9 activation, and tight-junction suppression	[35]
Metabolic/endocrine system (pancreas/glucose homeostasis)	Subchronic oral AlCl_3_-induced diabetogenic and pancreatic islet injury model	Adult male albino rats	10/group (20 total)	50 mg/kg/day AlCl_3_	Oral gavage	28 days	Hyperglycaemia and impaired glucose tolerance with pancreatic islet necrosis and reduced islet cell density	[36]
Musculoskeletal system (skeletal muscle)	Experimental AlCl_3_-induced skeletal muscle contractility and sarcoplasmic reticulum dysfunction study	*Rana temporaria* frog tibialis anterior muscle fascicles	n = 10 experimental replicates	10^−4^–10^−2^ M AlCl_3_ solutions	Direct ex vivo tissue exposure	Acute experimental exposure during muscle stimulation assays	Concentration-dependent suppression of skeletal muscle contraction and sarcoplasmic reticulum Ca^2+^/Mg^2+^-ATPase activity	[37]
Skeletal system (osteoblasts/bone)	AlCl_3_-induced osteoblast dysfunction model	Primary osteoblasts isolated from 3-day-old Wistar rats	10 samples/group	0.126 mg/mL AlCl_3_·6H_2_O	Direct in vitro exposure	24 h	Osteoblast dysfunction with suppression of osteogenic signaling pathways and ultrastructural degeneration	[38]

**Table 2:** Summary of key biomarkers, direction of change, analytical methods, and representative references used to assess Alzheimer-like neuropathology in aluminum chloride (AlCl_3_)-induced experimental models.

Category	Biomarker	Change	Method	Example Reference
Amyloid pathology	Aβ_1–42_	↑ levels in AlCl_3_-induced rats	Immunohistochemistry, Western blot	[18]
Amyloid pathology	Aβ1 peptide	↑ Aβ peptide in AlCl_3_-induced rat brain	ELISA	[51]
Tau pathology	Phosphorylated tau (p-tau181/p-tau231/p-tau217)	↑ levels/hyperphosphorylation in AD	Plasma/CSF immunoassays, PET/MRI correlation	[52]
Neuronal injury	NeuN/Nissl	↓ neuronal density/neuronal loss	Nissl staining, NeuN immunostaining	[53]
Synaptic integrity	Synaptophysin (Syp)	↓ Syp immunoreactivity/synaptic loss	Immunohistochemistry	[54]
Neuroinflammation	GFAP, Iba-1	↑ astrocyte and microglial activation	Immunohistochemistry	[55]
Cytokine signaling	IL-1β, IL-6, TNF-α	↑ pro-inflammatory cytokines	ELISA	[7]
Oxidative stress	malondialdehyde (MDA), GSH, SOD, CAT	↑ lipid peroxidation (MDA); ↓ antioxidant enzymes (GSH, SOD, CAT)	Biochemical assays	[7]
Mitochondrial function	Mitochondrial complex I (NADH-ubiquinone oxidoreductase)	↓ complex I activity/mitochondrial dysfunction	Enzyme assay	[56]
Cholinergic dysfunction	Acetylcholinesterase (AChE)	↑ AChE activity in AlCl_3_-induced rats	Enzyme activity assay (Ellman method)	[51]

↑ Symbol refers to the increase of the mentioned markers, while ↓ refers to the decrease of them.

## Data Availability

No new data were created or analyzed in this study. Data sharing is not applicable.

## Abbreviations

The following abbreviations are used in this manuscript:

ACP	Acid phosphatase
ACh	Acetylcholine
AChE	Acetylcholinesterase
AD	Alzheimer’s disease
AD-MSC	Adipose-derived mesenchymal stem cell
AKAP4	A-kinase anchor protein 4
ALP	Alkaline phosphatase
ALT	Alanine aminotransferase
AlCl_3_	Aluminum chloride
AOPP	Advanced oxidation protein products
AST	Aspartate aminotransferase
ATP	Adenosine triphosphate
ATPase	Adenosine triphosphatase
BAPTA-AM	1,2-bis(o-aminophenoxy)ethane-N,N,N′,N′-tetraacetic acid tetrakis(acetoxymethyl ester)
BDNF	Brain-derived neurotrophic factor
BMP-2	Bone morphogenetic protein-2
BUN	Blood urea nitrogen
CAT	Catalase
Cbfα1	Core-binding factor alpha 1
CC BY	Creative Commons Attribution
CD117	Cluster of differentiation 117
CHOP	C/EBP homologous protein
CLD1	Claudin-1
CSF	Cerebrospinal fluid
Cyp19a1	Cytochrome P450 family 19 subfamily A member 1
DAO	Diamine oxidase
DNA	Deoxyribonucleic acid
ELISA	Enzyme-linked immunosorbent assay
ER	Endoplasmic reticulum
ERK1/2	Extracellular signal-regulated kinase 1/2
FASL	Fas ligand
FSH	Follicle-stimulating hormone
Fe/Zn/Cu	Iron/zinc/copper
GFAP	Glial fibrillary acidic protein
GGT	Gamma-glutamyl transferase
GIT	Gastrointestinal tract
GLP-1	Glucagon-like peptide-1
GLUT4	Glucose transporter type 4
GPx	Glutathione peroxidase
GR	Glutathione reductase
GRP78	Glucose-regulated protein 78
GSH	Glutathione
GSK3β	Glycogen synthase kinase-3 beta
H_2_O_2_	Hydrogen peroxide
HDL	High-density lipoprotein
HOMA-β	Homeostatic model assessment of beta-cell function
HOMA-IR	Homeostatic model assessment of insulin resistance
HPT	Hypothalamic-pituitary-testicular
HT-29	Human colorectal adenocarcinoma cell line HT-29
Iba-1	Ionized calcium-binding adapter molecule 1
IGF-1	Insulin-like growth factor-1
IGF-I	Insulin-like growth factor-I
IL-1β	Interleukin-1 beta
IL-6	Interleukin-6
IL-10	Interleukin-10
IL-18	Interleukin-18
IRF8	Interferon regulatory factor 8
IRS-1	Insulin receptor substrate-1
JNK	c-Jun N-terminal kinase
KIM-1	Kidney injury molecule-1
Ki-67	Marker of proliferation Ki-67
LDH	Lactate dehydrogenase
LDL	Low-density lipoprotein
LH	Luteinizing hormone
LTP	Long-term potentiation
MDA	Malondialdehyde
MMP9	Matrix metalloproteinase-9
mRNA	Messenger ribonucleic acid
NADH	Nicotinamide adenine dinucleotide, reduced form
NAG	N-acetyl-beta-D-glucosaminidase
NF-κB	Nuclear factor kappa B
NGAL	Neutrophil gelatinase-associated lipocalin
NPSH	Non-protein thiols
Nrf2	Nuclear factor erythroid 2-related factor 2
OAZ3	Ornithine decarboxylase antizyme 3
OCLN	Occludin
ODF1	Outer dense fiber protein 1
PET	Positron emission tomography
PI3K	Phosphoinositide 3-kinase
PINK1	PTEN-induced kinase 1
ROS	Reactive oxygen species
Runx2	Runt-related transcription factor 2
SDH	Succinate dehydrogenase
Ser307	Serine 307
Ser473	Serine 473
Smad	Small mothers against decapentaplegic
SOD	Superoxide dismutase
Syp	Synaptophysin
T_3_	Triiodothyronine
T_4_	Thyroxine
TGF-β	Transforming growth factor beta
TGF-β1	Transforming growth factor beta 1
TNF-α	Tumor necrosis factor alpha
TSH	Thyroid-stimulating hormone
UPPC	University of Petra Pharmaceutical Center
Wnt3a	Wingless-related integration site family member 3A
XBP1	X-box binding protein 1
ZO-1	Zonula occludens-1

## References

[1] Willhite C.C., Karyakina N.A., Yokel R.A., Yenugadhati N., Wisniewski T.M., Arnold I.M., Momoli F., Krewski D. (2014). Systematic review of potential health risks posed by pharmaceutical, occupational and consumer exposures to metallic and nanoscale aluminum, aluminum oxides, aluminum hydroxide and its soluble salts. Crit. Rev. Toxicol..

[2] Alhusban A.A., Al-Azzeh G.I., Tarawneh O.A., Abuzaid H.M., Ata S.A. (2025). The Safety Assessment of Trace Elements in Omega-3 Fish Oil Products Commonly Used for Infants in Jordan. Palest. Med. Pharm. J..

[3] Martinez C.S., Piagette J.T., Escobar A.G., Martín Á., Palacios R., Peçanha F.M., Vassallo D.V., Exley C., Alonso M.J., Miguel M. (2017). Aluminum exposure at human dietary levels promotes vascular dysfunction and increases blood pressure in rats: A concerted action of NAD(P)H oxidase and COX-2. Toxicology.

[4] Kandimalla R., Vallamkondu J., Corgiat E.B., Gill K.D. (2016). Understanding aspects of Aluminum exposure in Alzheimer’s disease development. Brain Pathol..

[5] Bhargava V.P., Netam A.K., Singh R., Sharma P. (2021). Aluminium and neuro-degeneration: Mechanism of pathogenesis and possible strategies for mitigation. Asian J. Pharm. Res. Health Care.

[6] Hayat M., Khola N.U.H., Ahmed T. (2025). A systematic review of preclinical studies investigating the effects of pharmacological agents on learning and memory in prolonged aluminum-exposure-induced neurotoxicity. Brain Sci..

[7] Kazmi I., Afzal M., Imam F., Alzarea S.I., Patil S., Mhaiskar A., Shah U., Almalki W.H. (2024). Barbaloin’s chemical intervention in aluminum chloride induced cognitive deficits and changes in rats through modulation of oxidative stress, cytokines, and BDNF expression. ACS Omega.

[8] Oyagbemi A.A., Femi-Akinlosotu O.M., Obasa A.A., Ojo M.S., Salami A.T., Ajibade T.O., Onukak C.E., Igado O.O., Esan O.O., Oyagbemi T.O. (2025). Apigenin mitigates oxidative stress, neuroinflammation, and cognitive impairment but enhances learning and memory in aluminum chloride-induced neurotoxicity in rats. Alzheimer’s Dement..

[9] Nafea M., Elharoun M., Abd-Alhaseeb M.M., Helmy M.W. (2023). Leflunomide abrogates neuroinflammatory changes in a rat model of Alzheimer’s disease: The role of TNF-α/NF-κB/IL-1β axis inhibition. Naunyn-Schmiedeberg’s Arch. Pharmacol..

[10] Lin W.-T., Chen R.-C., Lu W.-W., Liu S.-H., Yang F.-Y. (2015). Protective effects of low-intensity pulsed ultrasound on aluminum-induced cerebral damage in Alzheimer’s disease rat model. Sci. Rep..

[11] El-Ganainy S.O., Soliman O.A., Ghazy A.A., Allam M., Elbahnasi A.I., Mansour A.M., Gowayed M.A. (2022). Intranasal oxytocin attenuates cognitive impairment, β-amyloid burden and tau deposition in female rats with Alzheimer’s disease: Interplay of ERK1/2/GSK3β/caspase-3. Neurochem. Res..

[12] Klotz K., Weistenhöfer W., Neff F., Hartwig A., van Thriel C., Drexler H. (2017). The health effects of aluminum exposure. Dtsch. Arztebl. Int..

[13] Cutipa-Díaz Y.M., Huanacuni-Lupaca C., Limache-Sandoval E.M., Mamani-Huanca D.Y., Sánchez-Esquiche W.M., Rubira-Otarola D.G., Gutiérrez-Cueva R.N., Sacari Sacari E.J. (2024). Exposure to aluminum in drinking water and the risk of developing alzheimer’s disease: A bibliometric analysis and systematic evaluation. Water.

[14] Narwanto M.I., Rahayu M., Soeharto S. (2022). Aluminum chloride impaired spatial memory, but not senile plaques formation in the rat model of Alzheimer’s disease. Sains Med. J. Med. Health.

[15] Martinez C.S., Alterman C.D., Peçanha F.M., Vassallo D.V., Mello-Carpes P.B., Miguel M., Wiggers G.A. (2017). Aluminum exposure at human dietary levels for 60 days reaches a threshold sufficient to promote memory impairment in rats. Neurotox. Res..

[16] Bittencourt L.O., Damasceno-Silva R.D., Aragão W.A.B., Eiró-Quirino L., Oliveira A.C.A., Fernandes R.M., Freire M.A.M., Cartágenes S.C., Dionizio A., Buzalaf M.A.R. (2022). Global proteomic profile of aluminum-induced hippocampal impairments in rats: Are low doses of aluminum really safe?. Int. J. Mol. Sci..

[17] Zhang L., Jin C., Liu Q., Lu X., Wu S., Yang J., Du Y., Zheng L., Cai Y. (2013). Effects of subchronic aluminum exposure on spatial memory, ultrastructure and L-LTP of hippocampus in rats. J. Toxicol. Sci..

[18] Singh N.A., Bhardwaj V., Ravi C., Ramesh N., Mandal A.K.A., Khan Z.A. (2018). EGCG nanoparticles attenuate aluminum chloride induced neurobehavioral deficits, beta amyloid and tau pathology in a rat model of Alzheimer’s disease. Front. Aging Neurosci..

[19] Sedik A.A., Hassan S.A., Shafey H.I., Khalil W.K., Mowaad N.A. (2023). Febuxostat attenuates aluminum chloride-induced hepatorenal injury in rats with the impact of Nrf2, Crat, Car3, and MNK-mediated apoptosis. Environ. Sci. Pollut. Res..

[20] Meliana A., Ratnani A.H.P., Hasanatuludhhiyah N., Rahniayu A., Mastutik G., Rahaju A.S. (2024). Protective effect of olive leaf (*Olea europaea* L.) extract against chronic exposure of liver and kidney tissues of Wistar rats to aluminum chloride. J. Herbmed Pharmacol..

[21] Alqhtani H.A. (2025). Evaluation of L-carnitine’s protective properties against AlCl_3_-induced brain, liver, and renal toxicity in rats. PLoS ONE.

[22] Hassan N.H., Yousef D.M., Alsemeh A.E. (2023). Hesperidin protects against aluminum-induced renal injury in rats via modulating MMP-9 and apoptosis: Biochemical, histological, and ultrastructural study. Environ. Sci. Pollut. Res..

[23] Kadhim A., Ben Slima A., Alneamah G., Makni M. (2024). Assessment of histopathological alterations and oxidative stress in the liver and kidney of male rats following exposure to aluminum chloride. J. Toxicol..

[24] Wei H., Li D., Luo Y., Wang Y., Lin E., Wei X. (2023). Aluminum exposure induces nephrotoxicity via fibrosis and apoptosis through the TGF-β1/Smads pathway in vivo and in vitro. Ecotoxicol. Environ. Saf..

[25] Tayo A.B., Abubakar J., Gulumbe B.H., Auwal A.R., Shitu A., Danjuma A.M. (2024). Cardio and neuroprotective effects of naringenin against aluminum chloride-induced oxidative stress in wistar rats. Avicenna J. Med. Biochem..

[26] Ghorbel I., Chaâbane M., Boudawara O., Kamoun N.G., Boudawara T., Zeghal N. (2016). Dietary unsaponifiable fraction of extra virgin olive oil supplementation attenuates lung injury and DNA damage of rats co-exposed to aluminum and acrylamide. Environ. Sci. Pollut. Res..

[27] Amin M., Saad S. (2017). A Study of the Effect of Aluminum Chloride on Pneumocyte Type II Cells of Albino Rats and Possible Protective Role of Propolis. Egypt. J. Anat..

[28] Peng H., Huang Y., Wei G., Pang Y., Yuan H., Zou X., Xie Y., Chen W. (2024). Testicular toxicity in rats exposed to AlCl_3_: A Proteomics Study. Biol. Trace Elem. Res..

[29] Abo El-Ela F.I., Gamal A., El-Banna H.A., Ibrahim M.A., El-Banna A.H., Abdel-Razik A.-R.H., Abdel-Wahab A., Hassan W.H., Abdelghany A.K. (2024). Repro-protective activity of amygdalin and spirulina platensis in niosomes and conventional forms against aluminum chloride–induced testicular challenge in adult rats: Role of CYP11A1, StAR, and HSD-3B expressions. Naunyn-Schmiedeberg’s Arch. Pharmacol..

[30] Mansour F.R., Nabiuni M., Amini E. (2022). Ovarian toxicity induced by aluminum chloride: Alteration of Cyp19a1, Pcna, Puma, and Map1lc3b genes expression. Toxicology.

[31] AL-Kaisei B.I., Humadai T.J., Alamaary A.N.F., Salih A.M.M. (2017). Toxicopathological Effects of Aluminum Chloride (AlCl_3_) in Reproductive System of Female Albino Mice. J. Kerbala Agric. Sci..

[32] Almarzany Z.S.K. (2020). Protective roles of melatonin on Hematological Parameters and Thyroid Hormone Levels in rats treated with Aluminum Chloride. Zanco J. Pure Appl. Sci..

[33] Al Nahari H., Al Eisa R. (2016). Effect of turmeric (*Curcuma longa*) on some pituitary, thyroid and testosterone hormone against aluminum chloride (AlCl_3_) induced toxicity in rat. Adv. Environ. Biol..

[34] Jeong C.H., Kwon H.C., Cheng W.N., Kang S., Shin D.-M., Yune J.H., Yoon J.E., Chang Y.H., Sohn H., Han S.G. (2020). Effects of aluminum on the integrity of the intestinal epithelium: An in vitro and in vivo study. Environ. Health Perspect..

[35] Hao W., Zhu X., Liu Z., Song Y., Wu S., Lu X., Yang J., Jin C. (2022). Resveratrol alleviates aluminum-induced intestinal barrier dysfunction in mice. Environ. Toxicol..

[36] Igwenagu E., Igbokwe I.O., Egbe-Nwiyi T.N. (2020). Fasting hyperglycaemia, glucose intolerance and pancreatic islet necrosis in albino rats associated with subchronic oral aluminium chloride exposure. Comp. Clin. Pathol..

[37] Nozdrenko D., Abramchuk O., Soroca V., Miroshnichenko N. (2015). Aluminum chloride effect on Ca^2+^, Mg^2+^-ATPase activity and dynamic parameters of skeletal muscle contraction. Ukr. Biochem. J..

[38] Zhu Y., Hu C., Zheng P., Miao L., Yan X., Li H., Wang Z., Gao B., Li Y. (2016). Ginsenoside Rb1 alleviates aluminum chloride-induced rat osteoblasts dysfunction. Toxicology.

[39] Armstrong R.A. (2019). Risk factors for Alzheimer’s disease. Folia Neuropathol..

[40] Todkar R., Shirote P., Mohite S. (2025). In Silico Screening and DFT Analysis of Nelumbo nucifera Phytochemicals as Potential BACE-1 Inhibitors for Alzheimer’s disease. Prospect. Pharm. Sci..

[41] Łysiak K., Łysiak A. (2024). The role of the gut microbiome in Alzheimer’s disease. Prospect. Pharm. Sci..

[42] Shahabuddin F., Naseem S., Alam T., Khan A.A., Khan F. (2024). Chronic aluminium chloride exposure induces redox imbalance, metabolic distress, DNA damage, and histopathologic alterations in Wistar rat liver. Toxicol. Ind. Health.

[43] Korotkov S.M. (2023). Mitochondrial oxidative stress is the general reason for apoptosis induced by different-valence heavy metals in cells and mitochondria. Int. J. Mol. Sci..

[44] Wong-Guerra M., Montano-Peguero Y., Ramírez-Sánchez J., Jiménez-Martin J., Fonseca-Fonseca L.A., Hernández-Enseñat D., Nonose Y., Valdés O., Mondelo-Rodriguez A., Ortiz-Miranda Y. (2021). Multifunctional molecule, JM-20, reverses aluminum chloride-induced memory impairment and neuronal damage in rats. Neurotoxicology.

[45] Abu-Zaid H. (2023). Toxic Metals Transfer from Heating Coils to e-liquids: Safety Assessment of Popular e-cigarettes in Jordan. Jordan J. Pharm. Sci..

[46] Umesalma S. (2015). Protective Effect of Centella asiatica against Aluminium-Induced Neurotoxicity in Cerebral Cortex, Striatum, Hypothalamus and Hippocampus of Rat Brain- Histopathological, and Biochemical Approach. J. Mol. Biomark. Diagn..

[47] Annita A., Revilla G., Ali H., Almurdi A. (2024). Adipose-derived mesenchymal stem cell (AD-MSC)-like cells restore nestin expression and reduce amyloid plaques in aluminum chloride (AlCl_3_)-driven Alzheimer’s rat models. Mol. Cell. Biomed. Sci..

[48] Sharma R.K. (2023). Role of Aluminium in Alzheimer’s disease: Ultrastructural Study in Rat Hippocampus. Int. J. Anat. Res..

[49] Mahmoud M.N., Mohamed D.A., Mohamed E.K., Bushra R.R. (2024). Potential Role of Bone Marrow Mesenchymal Stem Cells in Ameliorating Hippocampal Structural Changes Induced by Aluminium Chloride in Adult Male Albino Rats. Egypt. Acad. J. Biol. Sci. D. Histol. Histochem..

[50] Akiyama H., Hosokawa M., Kametani F., Kondo H., Chiba M., Fukushima M., Tabira T. (2012). Long-term oral intake of aluminium or zinc does not accelerate Alzheimer pathology in AβPP and AβPP/tau transgenic mice. Neuropathology.

[51] Li D., Wang M. (2024). Shikonin Attenuate Behavioral Defects, Oxidative Stress, and Neuroinflammation During Aluminum Chloride-induced Alzheimer’s Disease Condition in an in vivo Experimental Model. Pharmacogn. Mag..

[52] Karur P., Kaldas M., Ramesh Babu Y.S., Parmar M.S. (2026). Phosphorylated Tau Biomarkers in Alzheimer’s Disease: From Early Detection to Clinical Potential—A Comprehensive Review. Mol. Neurobiol..

[53] Zhang L., Li J., Lin A. (2021). Assessment of neurodegeneration and neuronal loss in aged 5XFAD mice. STAR Protoc..

[54] Asghar H., Siddiqui A., Batool L., Batool Z., Ahmed T. (2024). Post-exposure self-recovery reverses oxidative stress, ameliorates pathology and neurotransmitters imbalance and rescues spatial memory after time-dependent aluminum exposure in rat brain. Biometals.

[55] Shalaby A.M., Alnasser S.M., Khairy D.A., Alabiad M.A., Alorini M., Jaber F.A., Tawfeek S.E. (2023). The neuroprotective effect of ginsenoside Rb1 on the cerebral cortex changes induced by aluminium chloride in a mouse model of Alzheimer’s disease: A histological, immunohistochemical, and biochemical study. J. Chem. Neuroanat..

[56] Elariny H.A., Kabel A.M., Selim H.M.R.M., Helal A.I., Abdelrahman D., Borg H.M., Elkady M.A., Dawood L.M., El-Badawy M.F., Almalawi H.F.A. (2024). Repositioning Canagliflozin for Mitigation of Aluminium Chloride-Induced Alzheimer’s Disease: Involvement of TXNIP/NLRP3 Inflammasome Axis, Mitochondrial Dysfunction, and SIRT1/HMGB1 Signalling. Medicina.

[57] Majumdar A.S., Nirwane A., Kamble R. (2014). Coenzyme q10 abrogated the 28 days aluminium chloride induced oxidative changes in rat cerebral cortex. Toxicol. Int..

[58] Yuan C.-Y., Lee Y.-J., Hsu G.-S.W. (2012). Aluminum overload increases oxidative stress in four functional brain areas of neonatal rats. J. Biomed. Sci..

[59] Cheng H., Yang B., Ke T., Li S., Yang X., Aschner M., Chen P. (2021). Mechanisms of metal-induced mitochondrial dysfunction in neurological disorders. Toxics.

[60] Khan K., Emad N.A., Sultana Y. (2024). Inducing Agents for Alzheimer’s Disease in Animal Models. J. Explor. Res. Pharmacol..

[61] Promyo K., Iqbal F., Chaidee N., Chetsawang B. (2020). Aluminum chloride-induced amyloid β accumulation and endoplasmic reticulum stress in rat brain are averted by melatonin. Food Chem. Toxicol..

[62] Chiroma S.M., Baharuldin M.T., Mat Taib C.N., Amom Z., Jagadeesan S., Ilham Adenan M., Mahdi O., Moklas M.A. (2019). Centella asiatica Protects d-Galactose/AlCl_3_ Mediated Alzheimer’s Disease-Like Rats via PP2A/GSK-3β Signaling Pathway in Their Hippocampus. Int. J. Mol. Sci..

[63] Liang R.-f., Zhang H.-f., Wang H., Zhang Y., Niu Q. (2012). Aluminium-maltolate-induced impairment of learning, memory and hippocampal long-term potentiation in rats. Ind. Health.

[64] El-Shazly S.A., Alhejely A., Alghibiwi H.K., Dawoud S.F., Sharaf-Eldin A.M., Mostafa A.A., Zedan A.M., El-Sadawy A.A., El-Magd M.A. (2024). Protective effect of magnetic water against AlCl_3_-induced hepatotoxicity in rats. Sci. Rep..

[65] El-Demerdash F.M., Hussien D.M., Ghanem N.F., Al-Farga A.M. (2022). Bromelain modulates liver injury, hematological, molecular, and biochemical perturbations induced by aluminum via oxidative stress inhibition. BioMed Res. Int..

[66] Cheraghi E., Roshanaei K. (2019). The protective effect of curcumin against aluminum chloride-induced oxidative stress and hepatotoxicity in rats. Pharm. Biomed. Res..

[67] Al-Harbi M.S. (2019). Antioxidant, protective effect of black berry and quercetin against hepatotoxicity induced by aluminum chloride in male rats. Int. J. Pharmacol..

[68] Othman M.S., Fareid M.A., Abdel Hameed R.S., Abdel Moneim A.E. (2020). The protective effects of melatonin on aluminum-induced hepatotoxicity and nephrotoxicity in rats. Oxid. Med. Cell. Longev..

[69] Wei X., Luo Y., Yuan D., Li D., Nong Y., Wu B., Qin X. (2025). Effect of the Nrf2/HO-1 pathway on aluminum-induced liver injury. Ecotoxicol. Environ. Saf..

[70] Saljooghi A.S. (2012). Chelation of aluminum by combining deferasirox and deferiprone in rats. Toxicol. Ind. Health.

[71] Kunz S.N., Bohrer D., do Nascimento P.C., Cibin F.W.S., de Carvalho L.M. (2024). Interference of parenteral nutrition components in silicon-mediated protection against aluminum bioaccumulation. Biol. Trace Elem. Res..

[72] Spencer A., Wood J., Saunders H., Freeman M., Lote C. (1995). Aluminium deposition in liver and kidney following acute intravenous administration of aluminium chloride or citrate in conscious rats. Hum. Exp. Toxicol..

[73] Xia S., Li M., Shao B., Bai C., Zhang J., Li Y. (2013). Effects of sub-chronic aluminum exposure on renal pathologic structure in rats. J. Northeast Agric. Univ. (Engl. Ed.).

[74] Yavuz H., Şimşek H., Akaras N., Kandemir Ö., Tuncer S.Ç., Kandemir F.M. (2025). Protective role of catechin hydrate against aluminum chloride-induced nephrotoxicity via oxidative stress, NF-κB, Bax/Bcl-2, and PERK-CHOP pathways. Eur. J. Pharmacol..

[75] Al Kahtani M.A. (2019). Curcumin phytosome ameliorates aluminum chloride-induced nephrotoxicity in rats. Egypt. J. Hosp. Med..

[76] Al Dera H.S. (2016). Protective effect of resveratrol against aluminum chloride induced nephrotoxicity in rats. Saudi Med. J..

[77] Ghorbel I., Elwej A., Chaabane M., Jamoussi K., Zeghal N. (2017). Protective effect of selenium against aluminium chloride induced cardiotoxicity in rats. Pharm. Biomed. Res..

[78] Elsayed H.M., Mohammed W.I., Gebril S.M., Ahmed S.A. (2023). The Implication of Cardiac Telocytes, Inflammation, and Apoptosis in Carvedilol Protective Effect Against Aluminum Chloride Induced Myocardial Toxicity in Male Wistar Rats. Egypt. J. Histol..

[79] Monaco A., Grimaldi M., Ferrandino I. (2017). Aluminium chloride-induced toxicity in zebrafish larvae. J. Fish Dis..

[80] Hadrup N., Sørli J.B., Jenssen B.M., Vogel U., Sharma A.K. (2024). Toxicity and biokinetics following pulmonary exposure to aluminium (aluminum): A review. Toxicology.

[81] Ghorbel I., Elwej A., Chaabane M., Jamoussi K., Mnif H., Boudawara T., Zeghal N. (2017). Selenium alleviates oxidative stress and lung damage induced by aluminum chloride in adult rats: Biochemical and histological approach. Biol. Trace Elem. Res..

[82] El_Roghy E.S., Soliman M.E.-S., Atteya S., Zakaria H. (2022). Evaluation of propolis supplementation on lung tissue toxicity induced by aluminum chloride in adult male albino rats: A histological and immunohistochemical study. Egypt. J. Histol..

[83] Buraimoh A., Ojo S. (2013). Effects of aluminium chloride exposure on the histology of lungs of wistar rats. J. Appl. Pharm. Sci..

[84] Albambi E.-B. (2012). Effect of aluminum, cadmium and lead on rat lung: Protective role of selenium. Al-Azhar J. Pharm. Sci..

[85] Sulayman Alhasy Z., Maher I., Morsy M., Shehata M. (2020). Postnatal Changes of Lung Structure in Albino Rats after Aluminum Chloride Exposure and Possible Protective Role of Omega 3. Prensa Med. Argent..

[86] Nuhair R. (2015). Effects of Aluminum chloride on some hormones levele and reproductive organs of male rats (*Rattus norvegicus*). Univ. Thi-Qar J. Sci..

[87] Chen J., Xia Y., Ben Y., Lu X., Dou K., Ding Y., Han X., Yang F., Wang J., Li D. (2024). Embryonic exposure to aluminum chloride blocks the onset of spermatogenesis through disturbing the dynamics of testicular tight junctions via upregulating Slc25a5 in offspring. Sci. Total Environ..

[88] Nong W., Wei G., Wang J., Lei X., Wang J., Wei Y., Dong M., He L. (2024). Nicotinamide mononucleotide improves spermatogenic disorders in aluminum-exposed rats by modulating the glycolytic pathway. Biol. Trace Elem. Res..

[89] Peng H.-x., Chai F., Chen K.-h., Huang Y.-x., Wei G.-j., Yuan H., Pang Y.-f., Luo S.-h., Wang C.-f., Chen W.-c. (2024). Reactive oxygen species-mediated mitophagy and cell apoptosis are involved in the toxicity of aluminum chloride exposure in GC-2spd. Biol. Trace Elem. Res..

[90] Kalaiselvi A., Suganthy O.M., Govindassamy P., Vasantharaja D., Gowri B., Ramalingam V. (2014). Influence of aluminium chloride on antioxidant system in the testis and epididymis of rats. Iran. J. Toxicol..

[91] Lokman M., Ashraf E., Kassab R.B., Abdel Moneim A.E., El-Yamany N.A. (2022). Aluminum chloride–induced reproductive toxicity in rats: The protective role of zinc oxide nanoparticles. Biol. Trace Elem. Res..

[92] Boudou F., Bendahmane-Salmi M., Benabderrahmane M., Benalia A., Beghdadli B. (2020). The impact of aluminum chloride sub-acute exposure on the reproductive system of male rats. J. Exp. Res..

[93] Mohammed A.M., Al-Kaisei B.I., Humadai T.J., Al-Taee E.H. (2018). Effect of chronic aluminum chloride toxicity on sperm and reproductive markers in albino mice. Int. J. Biosci..

[94] Pandey G., Jain G. (2017). Aluminium chloride-induced testicular effects in rats: A histomorphometrical study. Asian J. Appl. Sci. Technol..

[95] Wang N., She Y., Zhu Y., Zhao H., Shao B., Sun H., Hu C., Li Y. (2012). Effects of subchronic aluminum exposure on the reproductive function in female rats. Biol. Trace Elem. Res..

[96] Japhet L.B., Oluwatunase G.O., Adejayan T.O. (2024). Ethanolic extract of *Xylopia aethiopica* attenuated aluminum-induced ovarian toxicity in adult female wistar rats. JBRA Assist. Reprod..

[97] Chinoy N., Patel T.N. (2001). Effects of sodium fluoride and aluminium chloride on ovary and uterus of mice and their reversal by some antidotes. Fluoride.

[98] Sirisha K.B., Prasunpriya N., Archana, Kalyani M. (2022). Ovarian oxidative stress response due to aluminium exposure in Wistar rats. Neuroquantology.

[99] Fu Y., Jia F., Wang J., Song M., Liu S., Li Y., Liu S., Bu Q. (2014). Effects of sub-chronic aluminum chloride exposure on rat ovaries. Life Sci..

[100] Japhet L.B., Anna I.C., Precious A.I. (2024). Investigation on the Effect of *Xylopia aethiopica* Ethanol Seed Extract on Aluminum Chloride induced Uterus and Gonadotropins Toxicity in Adult Female Wistar Rats. Int. J. Biomed. Sci..

[101] Mekkey A.M. (2021). Histological–Physiological study of thyroid gland in white male rats processing with aluminum chloride and treated with oil of *Nigella sativa*. J. Univ. Babylon Pure Appl. Sci..

[102] Orihuela D. (2011). Aluminium effects on thyroid gland function: Iodide uptake, hormone biosynthesis and secretion. J. Inorg. Biochem..

[103] Yu L., Wu J., Zhai Q., Tian F., Zhao J., Zhang H., Chen W. (2019). Metabolomic analysis reveals the mechanism of aluminum cytotoxicity in HT-29 cells. PeerJ.

[104] Wang B., Wu C., Cui L., Wang H., Liu Y., Cui W. (2022). Dietary aluminium intake disrupts the overall structure of gut microbiota in Wistar rats. Food Sci. Nutr..

[105] Martinez C.S., Uranga-Ocio J., Peçanha F.M., Vassalo D., Miguel M., Wiggers G.A. (2022). Egg White Hydrolysate as a new bioactive food ingredient in the prevention of gastrointestinal effects induced by aluminum exposure in rats. Acad. J. Health Sci..

[106] Nampoothiri M., Kumar N., Ramalingayya G.V., Kutty N.G., Krishnadas N., Rao C.M. (2017). Effect of insulin on spatial memory in aluminum chloride-induced dementia in rats. Neuroreport.

[107] Wei X., Wei H., Yang D., Li D., Yang X., He M., Lin E., Wu B. (2018). Effect of aluminum exposure on glucose metabolism and its mechanism in rats. Biol. Trace Elem. Res..

[108] Mandlem V., Ka V.S., Rao G.K., Mannec R., Kosurud R. (2021). Dulaglutide Improves Aluminum Chloride Induced Cognitive Dysfunction Diabetes Associated Alzheimer’s Rat Mode. Ann. Biol. Res..

[109] Atabi F., Moassesfar M., Nakhaie T., Bagherian M., Hosseinpour N., Hashemi M. (2025). A systematic review on type 3 diabetes: Bridging the gap between metabolic dysfunction and Alzheimer’s disease. Diabetol. Metab. Syndr..

[110] Saeed M.M. (2024). Repurposing dapagliflozin for Alzheimer’s disease: A mechanistic exploration. Future J. Pharm. Sci..

[111] Sun X., Wang H., Huang W., Yu H., Shen T., Song M., Han Y., Li Y., Zhu Y. (2017). Inhibition of bone formation in rats by aluminum exposure via Wnt/β-catenin pathway. Chemosphere.

[112] Cao Z., Fu Y., Sun X., Zhang Q., Xu F., Li Y. (2016). Aluminum trichloride inhibits osteoblastic differentiation through inactivation of Wnt/β-catenin signaling pathway in rat osteoblasts. Environ. Toxicol. Pharmacol..

[113] Li X., Han Y., Guan Y., Zhang L., Bai C., Li Y. (2012). Aluminum induces osteoblast apoptosis through the oxidative stress-mediated JNK signaling pathway. Biol. Trace Elem. Res..

[114] Cao Z., Liu D., Zhang Q., Sun X., Li Y. (2016). Aluminum chloride induces osteoblasts apoptosis via disrupting calcium homeostasis and activating Ca^2+^/CaMKII signal pathway. Biol. Trace Elem. Res..

[115] Zhu Y., Xu F., Yan X., Miao L., Li H., Hu C., Wang Z., Lian S., Feng Z., Li Y. (2016). The suppressive effects of aluminum chloride on the osteoblasts function. Environ. Toxicol. Pharmacol..

[116] Song M., Cui Y., Wang Q., Zhang X., Zhang J., Liu M., Li Y. (2021). Ginsenoside Rg3 alleviates aluminum chloride-induced bone impairment in rats by activating the TGF-β1/Smad signaling pathway. J. Agric. Food Chem..

[117] European Food Safety Authority (2008). Safety of aluminium from dietary intake-scientific opinion of the panel on food additives, flavourings, processing aids and food contact materials (AFC). EFSA J..

[118] Kongta N., Judprasong K., Chunhabundit R., Sirivarasai J., Karnpanit W. (2023). Assessment of Exposure to Aluminum through Consumption of Noodle Products. Foods.

[119] Weisser K. (2024). Toxicokinetics of aluminium—Novel insights in an old adjuvant. Allergo J. Int..

[120] Vlasak T., Dujlovic T., Barth A. (2024). Aluminum exposure and cognitive performance: A meta-analysis. Sci. Total Environ..

[121] Soleimani H., Dehghani S., Abolli S., Alamdari H.A., Gheisvandi O., Atlasi R., Yazdi N.B., Tabatabaei-Malazy O., Soleimani Z., Handy R.D. (2025). Environmental aluminum exposure and Alzheimer’s disease risk: Evidence from a systematic review and meta-analysis. Ecotoxicol. Environ. Saf..

[122] Zaitseva N.V., Zemlyanova M.A., Gekht A.B., Dedaev S.I., Kol’dibekova Y.V., Peskova E.V., Stepankov M.S., Tinkov A.A., Martins A.C., Skalny A.V. (2025). Neurotoxic effects of aluminum and manganese: From molecular to clinical effects. J. Neurol. Sci..

